# A PQM-1-Mediated Response Triggers Transcellular Chaperone Signaling and Regulates Organismal Proteostasis

**DOI:** 10.1016/j.celrep.2018.05.093

**Published:** 2018-06-26

**Authors:** Daniel O’Brien, Laura M. Jones, Sarah Good, Jo Miles, M.S. Vijayabaskar, Rebecca Aston, Catrin E. Smith, David R. Westhead, Patricija van Oosten-Hawle

**Affiliations:** 1 School of Molecular and Cell Biology and Astbury Centre for Structural Molecular Biology, University of Leeds, Leeds, UK

**Keywords:** proteostasis, *C. elegans*, transcellular chaperone signaling, stress response, HSP-90, PQM-1, CLEC-41, ASP-12

## Abstract

In metazoans, tissues experiencing proteotoxic stress induce “transcellular chaperone signaling” (TCS) that activates molecular chaperones, such as *hsp-90*, in distal tissues. How this form of inter-tissue communication is mediated to upregulate systemic chaperone expression and whether it can be utilized to protect against protein misfolding diseases remain open questions. Using *C. elegans*, we identified key components of a systemic stress signaling pathway that links the innate immune response with proteostasis maintenance. We show that mild perturbation of proteostasis in the neurons or the intestine activates TCS via the GATA zinc-finger transcription factor PQM-1. PQM-1 coordinates neuron-activated TCS via the innate immunity-associated transmembrane protein CLEC-41, whereas intestine-activated TCS depends on the aspartic protease ASP-12. Both TCS pathways can induce *hsp-90* in muscle cells and facilitate amelioration of *Aβ*_*3-42*_-associated toxicity. This may have powerful implications for the treatment of diseases related to proteostasis dysfunction.

## Highlights

• PQM-1 is activated in the neurons or gut to induce TCS-mediated *hsp-90* expression
• Neuron-induced TCS is mediated via PQM-1/CLEC-41 signaling
• Intestine-induced TCS is mediated via PQM-1/ASP-12 signaling
• TCS via PQM-1 is required for proteostasis and reduces amyloid beta misfolding

## Graphical Abstract

**Figure:**
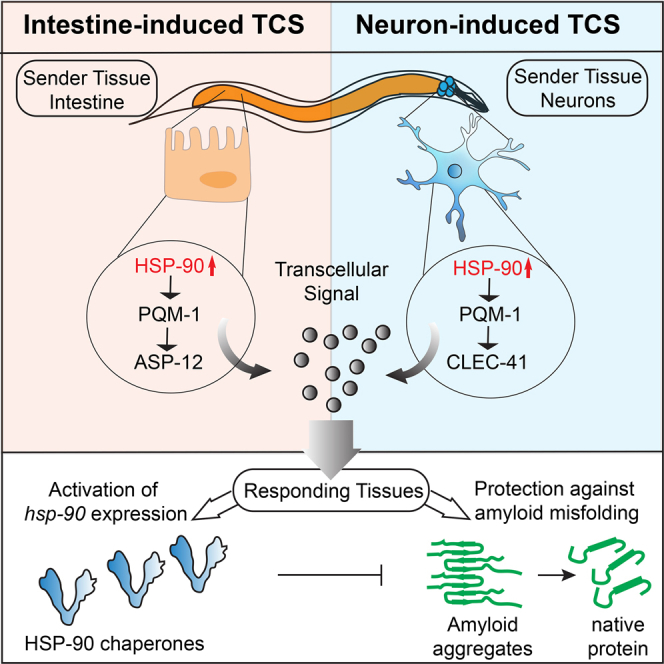

## Teaser

O’Brien et al. find that the GATA transcription factor PQM-1 functions as a mediator of transcellular chaperone signaling (TCS). Depending on the sender tissue, PQM-1 activates a neuron-specific or intestine-specific TCS route that triggers *hsp-90* expression in remote tissues. TCS-mediated *hsp-90* induction reduces amyloid beta oligomerization and toxicity.

## Introduction

All living systems are confronted with a broad range of environmental insults and physiological changes that can result in cellular stress and macromolecular damage (Morimoto et al., 1997). To re-establish protein homeostasis or proteostasis, each cell has the ability to rapidly activate stress response pathways. These include the heat shock factor-1 (HSF-1)-mediated heat shock response (HSR), the unfolded protein response (UPR), and the insulin-like-signaling pathway (ILS) mediated by the FOXO/DAF-16 transcription factor (Gardner et al., 2013, Hsu et al., 2003, Morimoto et al., 1997). Stress response pathways upregulate cell-autonomous modifiers of the proteostasis network (PN), such as molecular chaperones, that protect protein conformation during stress, refold misfolded proteins, and target irreversibly misfolded proteins to the degradation machinery (Akerfelt et al., 2010, Morimoto et al., 1997).

In addition to stress response cues that act locally and within the cell, multicellular organisms also require intercellular stress response pathways that allow for coordinated activation of protective PN components across tissues to preserve proteostasis in the entire organism. In *C. elegans*, this is achieved by activation of the HSR via the thermo-sensory neuronal circuitry in response to heat stress (Prahlad et al., 2008). In response to stressors of intracellular origin, such as perturbed mitochondrial function, DNA damage, and altered chaperone expression in specific tissues, a systemic stress signaling response is activated that promotes PN components in cells distinct from the stressed sender cell (Chikka et al., 2016, Durieux et al., 2011, Ermolaeva et al., 2013, van Oosten-Hawle and Morimoto, 2014, Prahlad and Morimoto, 2011, Taylor et al., 2014, Taylor and Dillin, 2013). Transcellular regulation of protective stress responses is not unique to the metazoan system of *C. elegans* and has been observed in other model systems, including vertebrates (Mahadevan et al., 2011, Owusu-Ansah et al., 2013, Williams et al., 2014). Understanding how the PN coordinates the activation of protective components in a systemic manner is fundamentally important.

Transcellular chaperone signaling (TCS) can be activated by overexpression of the essential molecular chaperone heat shock protein 90 (HSP-90) in specific tissues. This generates a form of tissue-specific proteostatic imbalance that induces non-cell-autonomous *hsp-90* expression and suppresses misfolding of metastable clients in remote tissues (van Oosten-Hawle et al., 2013). In this study, we investigated the central question of how non-cell-autonomous regulation of *hsp-90* chaperone expression is achieved and whether it can be utilized to protect from the toxic consequences of aggregation-prone human disease proteins. Which signaling cues are activated in the “sender” cell to initiate TCS-mediated protective mechanisms in a distal tissue affected by protein misfolding disease?

We identified key components of TCS that are, depending on the sender tissue, differentially regulated by the transcription factor PQM-1. When initiated from the neurons, PQM-1 orchestrates TCS via the innate immunity-associated transmembrane protein CLEC-41, whereas TCS initiated from the intestine depends on the aspartic protease ASP-12. Both TCS pathways mediate “transcellular” *hsp-90* induction in the muscle and protect against muscle-expressed amyloid protein misfolding.

## Results

### Increased Neuron- and Intestine-Specific Expression of HSP-90 Activates TCS and Suppresses Aβ_3-42_ Toxicity in Muscle Cells

**Figure 1:**
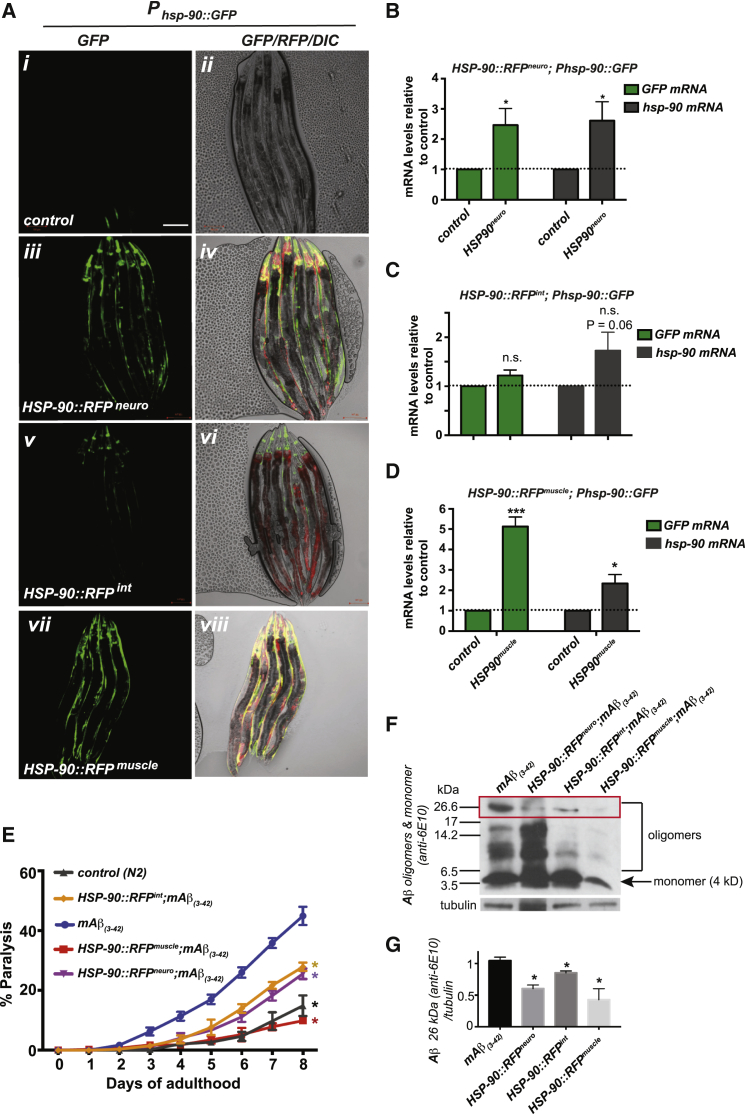
Increased Expression of HSP-90 in the Neurons, Intestine, or Bodywall Muscle Activates TCS and Protects against Aβ_(3-42)_ Protein Toxicity in Muscle (A–D) *P*_*hsp-90::*_*GFP* expression in control animals (*i* and *ii*), *HSP-90::RFP*^*neuro*^ (*iii* and *iv*), *HSP-90::RFP*^*int*^ (*v* and *vi*), or *HSP-90::RFP*^*muscle*^ (*vii* and *viii*) (A). Scale bar, 50 μm. GFP and *hsp-90* mRNA levels in *HSP-90::RFP*^*neuro*^*; P*_*hsp-90*_*::GFP* (B), *HSP-90::RFP*^*int*^*; P*_*hsp-90*_*::GFP* (C), and *HSP-90::RFP*^*muscle*^*; P*_*hsp-90*_*::GFP* (D) relative to control animals expressing *P*_*hsp-90*_*::GFP*. (B–D) Bar graphs represent the mean of three independent experiments. Error bars represent ± SEM. ^∗^p < 0.05, ^∗∗∗^p < 0.001; ns, not significant. (E) Paralysis assays of *C. elegans* expressing Aβ_(3-42)_ in the bodywall muscle (CL2006) compared to wild-type (N2) or *HSP-90::RFP*^*neuro*^*;Aβ*_*(3-42)*_, *HSP-90::RFP*^*int*^*;Aβ*_*(3-42)*_, or *HSP-90::RFP*^*muscle*^*;Aβ*_*(3-42)*_. Paralysis data represent SEM of 3 biological replicates (n = 100 animals). Statistical significance was determined by Wilcoxon matched-pairs signed-rank test. ^∗^p < 0.05. (F) Western blot analysis of amyloid beta monomeric and oligomeric amyloid beta expression in day 3 adult control animals (*mAβ*_*(3-42*_), *HSP-90::RFP*^*neuro*^*;Aβ*_*(3-42)*_, *HSP-90::RFP*^*int*^*;Aβ*_*(3-42)*_, or *HSP-90::RFP*^*muscle*^*;Aβ*_*(3-42)*_. (G) Quantification of oligomers (band at 26.6 kDa) in *mAβ*_*(3-42)*_, compared with *HSP-90::RFP*^*neuro*^*;Aβ*_*(3-42)*_, *HSP-90::RFP*^*int*^*;Aβ*_*(3-42)*_, or *HSP-90::RFP*^*muscle*^*;Aβ*_*(3-42)*_. Data are expressed as mean density of the indicated band based from three independent experiments. Error bars represent SEM. One-way ANOVA; ^∗^p < 0.05. See also Figure S1.

Activation of TCS in neurons, intestine or muscle cells by expressing elevated HSP-90::mCherry protein in these tissues (henceforth called *HSP-90::RFP*^*neuro*^*; HSP-90::RFP*^*int*^ or *HSP-90::RFP*^*muscle*^) leads to the non-cell-autonomous induction of a transcriptional *hsp-90* reporter fused to GFP (*P*_*hsp-90*_*::GFP*) (Figure 1A). In *HSP-90::RFP*^*neuro*^, this corresponded to a 30% increase of HSP-90::RFP protein present in the neurons relative to whole-animal endogenous HSP-90 levels (Figures S1A and S1B). This resulted in a 2.5-fold induction of *GFP* mRNA levels of the transcriptional *P*_*hsp-90*_*::GFP* reporter and global *hsp-90* mRNA levels, as measured by qRT-PCR (Figure 1B). In comparison, activation of TCS by expressing a 51% increase of HSP-90::RFP protein in the intestine (Figures S1A and S1B) induced GFP expression 1.3-fold (not significant [ns]) and *hsp-90* levels 1.75-fold (p = 0.06) (Figure 1C). In *HSP-90::RFP*^*muscle*^, a 82% increase of HSP-90::RFP relative to endogenous levels (Figures S1A and S1B) led to the highest induction of the transcriptional *P*_*hsp-90*_*::GFP* reporter resulting in 5-fold increased expression of *GFP* mRNA and a 2-fold induction of *hsp-90* mRNA levels (Figure 1D). Induction of *P*_*hsp-90*_*::GFP* transcriptional activity and *hsp-90* mRNA levels was significantly higher in *HSP-90::RFP*^*neuro*^ (Figures 1A–1D), despite the fact that the expression level of HSP-90::RFP protein in *HSP-90::RFP*^*neuro*^ is lower than *HSP-90::RFP*^*int*^ or *HSP-90::RFP*^*muscle*^ (Figures S1A and S1B).

**Figure 2:**
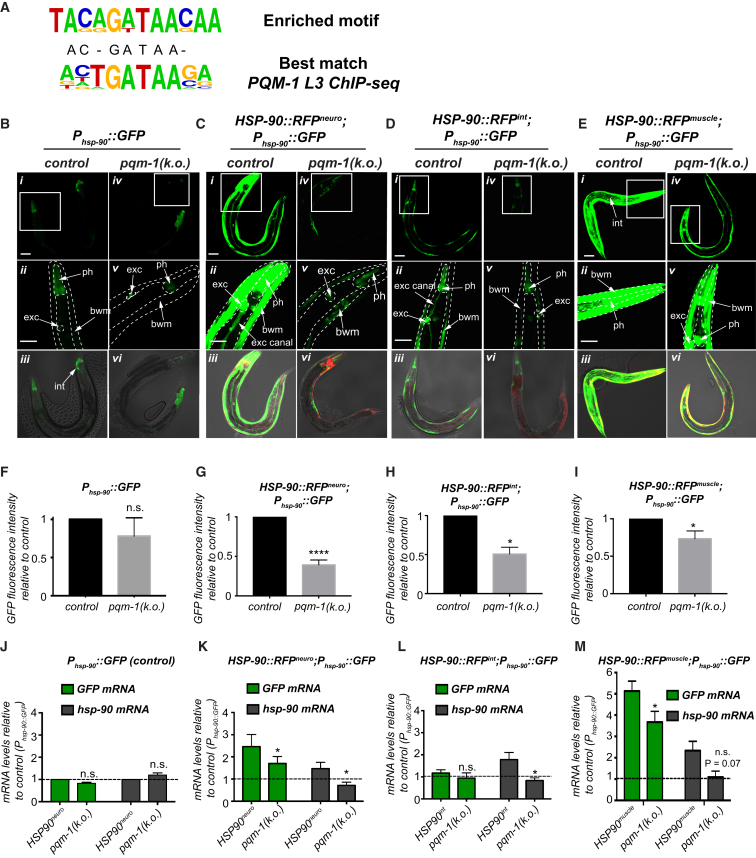
The Zinc-Finger Transcription Factor PQM-1 Mediates TCS in *C. elegans* (A) *Ab initio* motif discovery with HOMER identifies a significantly enriched motif (GATAA) in the promoters of 27 of the 34 genes upregulated in *HSP-90::GFP*^*neuro*^ compared to control (N2). (B–E) Collapsed z stack images of young adult *C. elegans* expressing the *P*_*hsp-90*_*::GFP* reporter in control background (B*i*), *HSP-90::RFP*^*neuro*^ (C*i*), *HSP-90::RFP*^*int*^ (D*i*), and *HSP-90::RFP*^*muscle*^ (E*i*) compared with expression of the *P*_*hsp-90*_*::GFP* reporter in a *pqm-1(KO)* mutant background. Expression of *P*_*hsp-90*_*::GFP* in a *pqm-1(KO)* mutant background (B*iv*), *HSP-90::RFP*^*neuro*^;*pqm-1 (KO)* (C*iv*), *HSP-90::RFP*^*int*^*;pqm-1(KO)* (D*iv*), or *HSP-90::RFP*^*muscle*^ (E*iv*). 20× magnification of the anterior (head) region (B*ii*, B*v*, C*ii,* C*v*, D*ii*, D*v*, E*ii*, and E*v*). Differential interference contrast (DIC) Nomarski, GFP, RFP overlay images. Tissues showing *P*_*hsp-90*_*::GFP* expression are indicated with a white arrow. bwm, bodywall muscle; exc, excretory cell; exc canal, excretory canal; ph, pharynx; and int, intestine. (F–I) Quantification of GFP fluorescence intensity in *C. elegans* expressing *P*_*hsp-90*_*::GFP* in the *pqm-1(KO)* mutant compared to control background (F), *HSP-90::RFP*^*neuro*^;*pqm-1(KO)* (G), or *HSP-90::RFP*^*int*^;*pqm-1(KO)* (H), or *HSP-90::RFP*^*muscle*^;*pqm-1(KO)* (I) relative to control. (J–M) *GFP* and *hsp-90* mRNA levels are reduced in *HSP-90::GFP*^*neuro*^*;P*_*hsp-90*_*::GFP*;*pqm-1(KO)* (K), *HSP-90::GFP*^*int*^*;P*_*hsp-90*_*::GFP;pqm-1(KO)* (L), and *HSP-90::GFP*^*muscle*^*;P*_*hsp-90*_*::GFP;pqm-1(KO)* (M)*,* but not in *P*_*hsp-90*_*::GFP*;*pqm-1(KO)* (J) relative to the *P*_*hsp-90*_*::GFP* control strain. Scale bar, 50 μm (B–E). Bar graphs represent the mean of three independent experiments (F–M). Error bars represent ± SEM. ^∗^p < 0.05. ns, not significant. See also Figure S2.

At the level of individual tissues, *P*_*hsp-90*_*::GFP* reporter expression in control animals was primarily detected in pharyngeal tissue and some cells of the intestine during normal growth conditions (20°C) (Figures 2B*i–iii*). In *HSP-90::RFP*^*neuro*^ and *HSP-90::RFP*^*int*^, expression of *P*_*hsp-90*_*::GFP* was particularly increased in the bodywall muscle, pharynx, and excretory cell and canal, albeit to a lower extent in *HSP-90::RFP*^*int*^ when compared to *HSP-90::RFP*^*neuro*^ (Figures 2C*i–iii* and 2D*i–iii*). In the *HSP-90::RFP*^*muscle*^ strain, *P*_*hsp-90*_*::GFP* expression was strongly induced in the bodywall muscle, indicating a strong cell-autonomous induction of *hsp-90*, in addition to non-cell-autonomous induction of the reporter in pharynx, excretory cell, and intestine (Figures 2E*i*–2E*iii*).

### Neuron-Specific and Intestine-Specific Overexpression of HSP-90 Suppresses Aβ_3-42_ Toxicity in Muscle Cells via TCS

In *C. elegans*, expression of human Aβ_3-42_ in the bodywall muscle leads to age-dependent aggregation and toxicity of the protein, resulting in decreased motility and paralysis (Link, 1995, McColl et al., 2012). Amyloid beta (Aβ)−associated toxicity can be suppressed by PN components (Cohen et al., 2009, Morley et al., 2002), and depends on *hsp-90* expression (Brehme et al., 2014). We therefore asked whether the TCS-mediated upregulation of *hsp-90* in the bodywall muscle in *HSP-90::RFP*^*neuro*^ and *HSP-90::RFP*^*int*^ would suppress the toxic consequences of human Alzheimer’s Aβ protein misfolding.

We used a *C. elegans* model for Alzheimer’s disease, which expressed the cytotoxic human beta amyloid protein (mAβ_3-42_) in the *C. elegans* bodywall muscle (Link, 1995), leading to increased paralysis with age (Figure 1E). Importantly, overexpression of HSP-90::RFP in muscle, intestine, or neurons alone had no effect on motility throughout aging compared to control (N2) animals (Figure S1D). *HSP-90::RFP*^*muscle*^ completely abrogated *mAβ*_*3-42*_-associated paralysis resulting in animals that behaved similar to control animals (Figure 1E). *HSP-90::RFP*^*neuro*^ significantly suppressed *Aβ*_*3-42*_-dependent muscle dysfunction, with only 25% of *HSP-90::RFP*^*neuro*^*;mAβ*_*3-42*_ animals being paralyzed at day 8 of adulthood compared to 45% same age *mAβ*_*3-42*_ control animals (Figure 1E). Surprisingly, increased expression of *HSP-90::RFP*^*int*^ led to an equal level of suppression of *mAβ*_*3-42*_-associated toxicity, with 26% of animals being paralyzed at day 8 of adulthood (Figure 1E).

Intrigued by this observation, we determined whether suppression of paralysis in all three HSP-90 overexpression strains was associated with a decrease of toxic *Aβ*_*3-42*_ conformational variants. *HSP-90::RFP*^*muscle*^ diminished the levels of *Aβ* monomer and oligomeric species, consistent with the strongly reduced paralysis in this strain (Figures 1E and 1F), which is likely a cell-autonomous consequence of *HSP-90::RFP*^*muscle*^ overexpression. In *HSP-90::RFP*^*neuro*^ and *HSP-90::RFP*^*int*^, accumulation of monomer and smaller oligomeric species (<17 kDa) is clearly present; however, the accumulation of higher oligomeric species (26 kDa) was reduced to 60% in *HSP-90::RFP*^*neuro*^ and to 85% in *HSP-90::RFP*^*int*^, compared with a 58% reduction in *HSP-90::RFP*^*muscle*^ (Figure 1G). Thus, the significant reduction of higher oligomeric species (26 kDa) in all three HSP-90 overexpression strains likely contributes to the suppression of *Aβ* toxicity.

### The Transcription Factor PQM-1 Mediates TCS and Triggers Innate Immune Gene Expression

To investigate the underlying mechanism that mediates TCS upon HSP-90 overexpression, we performed RNA sequencing (RNA-seq) analyses using *HSP-90::GFP*^*neuro*^ animals that express a 46% increase of HSP-90::GFP (Figures S1A and S1B). RNA-seq analysis identified 225 genes differentially expressed in *HSP-90::GFP*^*neuro*^ relative to N2 control animals (Table S1) of which 34 genes (excluding 5 pseudogenes) were significantly upregulated >1.5-fold (p-adj < 0.05) (Table S2). Among these, the gene ontology (GO) term associated with innate immune responses was overrepresented (19 out of 34 genes) (Table S2). Confirmation of gene expression levels by qRT-PCR in *HSP-90::GFP*^*neuro*^ revealed generally lower levels of expression (Figure S1C), but significant correlation between the qRT-PCR and RNA-seq measurements (r = 0.55, p = 0.001). Expression levels of the same genes in *HSP-90::RFP*^*neuro*^ were comparable to those measured in *HSP-90::GFP*^*neuro*^ (r = 0.62; p = 0.00008) (Figure S1C), indicating that a common transcriptional program is induced upon neuronal HSP-90 overexpression. We performed hypergeometric optimization of motif enrichment (HOMER) analysis on the upregulated genes which identified a motif with the consensus AGATAACA or TGTTATCT enriched in the promoter regions in 27 of the 34 upregulated genes in *HSP-90::GFP*^*neuro*^ (p = 1 × 10^−9^, found in 28% of targets vs. 0.16% of background) (Figure 2A). The motif resembled the GATA-like DAE (DAF-16-associated element) which has been previously identified as the binding site for the C2H2 zinc-finger transcription factor PQM-1 (Tepper et al., 2013). Consistent with our finding, a previous PQM-1 chromatin immunoprecipitation sequencing (ChIP-seq) analysis confirms binding of PQM-1 to 16 promoters of genes identified in our dataset (see Table S2) (Niu et al., 2011).

To determine whether PQM-1 is involved in TCS, we used a *pqm-1(ok485)* knockout mutant (*pqm-1*(*KO*)), which contains a deletion mutation to remove exons 2 to 6, effectively depleting expression of *pqm-1* transcripts (Figure S1E) (Dowen et al., 2016, Tepper et al., 2013). Depletion of *pqm-1* in *C. elegans* expressing *HSP-90::RFP*^*neuro*^;*P*_*hsp-90*_*::GFP* reduced GFP fluorescence intensity to 40% (Figures 2C, 2G, and S2B) and to 50% in *HSP-90::RFP*^*int*^;*P*_*hsp-90*_*::GFP* (Figures 2D, 2H, and S2C), whereas depletion of *pqm-1* in *HSP-90::RFP*^*muscle*^;*P*_*hsp-90*_*::GFP* reduced GFP fluorescence to 75% (Figures 2E, 2I, and S2D), suggesting that *pqm-1* mediates TCS. However, absence of *pqm-1* in the *P*_*hsp-90*_*::GFP* control strain did not affect GFP fluorescence intensity (Figures 2B, 2F, and S2A).

At the level of individual tissues, *pqm-1(KO)* in *HSP-90::RFP*^*neuro*^ and *HSP-90::RFP*^*int*^ decreased *P*_*hsp-90*_*::GFP* expression in excretory cell and canal, pharynx, and bodywall muscle (Figures 2C and 2D, respectively), whereas in *HSP-90::RFP*^*muscle*^ this led to a visibly decreased expression of the reporter in pharynx and intestine (Figure 2E). *Pqm-1(KO)* in all three TCS-activated strains resulted in a general reduction of *hsp-90* mRNA levels (Figures 2K, 2L, and 2M), reducing global *hsp-90* expression back to the basal levels of the control strain (Figure 2J). The *pqm-1(KO)* mutation did not affect *hsp-90* or *GFP* mRNA expression levels in the control strain (Figure 2J), suggesting that *pqm-1* does not regulate *hsp-90* expression under normal growth conditions. Consistently, knockdown of *pqm-1* by RNAi in the control strain (Figures S2E and S2I), *HSP-90::RFP*^*neuro*^;*P*_*hsp-90*_*::GFP* (Figures S2F and S2J), in *HSP-90::RFP*^*int*^;*P*_*hsp-90*_*::GFP* (Figures S2G and S2K) or *HSP-90::RFP*^*muscle*^;*P*_*hsp-90*_*::GFP* (Figures S2H and S2L) phenocopied the effect of the *pqm-1(KO)* mutation on *P*_*hsp-90*_*::GFP* reporter and global *hsp-90* mRNA levels.

RNAi-mediated knockdown of *hsf-1* or *daf-16* had no effect on GFP fluorescence intensity of the reporter or *GFP* and *hsp-90* mRNA levels in all three TCS-activated strains, whereas *hsp-90* expression in the control strain was dependent on *hsf-1* or *daf-16* (Figure S3), confirming that neither DAF-16 nor HSF-1 are involved in TCS (van Oosten-Hawle et al., 2013).

The motif enrichment analysis also identified the FoxA transcription factor PHA-4 (ranked at position 13, p = 1e-5), previously shown to be required for TCS in *C. elegans* (van Oosten-Hawle et al., 2013). However, the PHA-4 consensus sequence was found in the promoter of only one putative target gene (*Y41C4A.11*) (Table S2). To investigate whether *pqm-1* and *pha-4* could act in the same genetic pathway to regulate TCS-induced *hsp-90* expression, we knocked down *pha-4* by RNAi in addition to *pqm-1(KO)*, which further reduced *GFP* and *hsp-90* mRNA levels in all three TCS-activated strains, indicating that *pqm-1* and *pha-4* may control TCS via two distinct pathways.

As *pqm-1* mediates TCS in response to tissue-specific HSP-90 overexpression, we sought to determine whether this was also the case when *hsp-90* is constitutively knocked down (KD) via tissue-specific *hsp-90* hairpin RNAi in the neurons (*hsp-90*^*neuro*^*(KD))* or intestine (*hsp-90*^*int*^*(KD)),* which leads to systemic upregulation of *hsp-70 (C12C8.1)* (Figure S4A) (van Oosten-Hawle et al., 2013). However, the absence of *pqm-1* had no effect on *hsp-70* mRNA expression in *hsp-90*^*neuro*^*(KD)* and *hsp-90*^*int*^*(KD)* (Figure S4A), and *pqm-1* mRNA levels were not affected by tissue-specific knockdown of *hsp-90* (Figure S4B). Thus, PQM-1 mediates TCS specifically in response to tissue-specific HSP-90 overexpression in the neurons or intestine, but not in response to reduced expression of *hsp-90* in the same tissues.

### PQM-1 Is Required for Stress-Induced *hsp-90* Expression and Heat Stress Resistance

PQM-1 complements DAF-16/FOXO to regulate longevity in *C. elegans* (Tepper et al., 2013). It is, however, unclear whether PQM-1 is involved in the regulation of cell stress responses or the maintenance of proteostasis in general.

**Figure 3:**
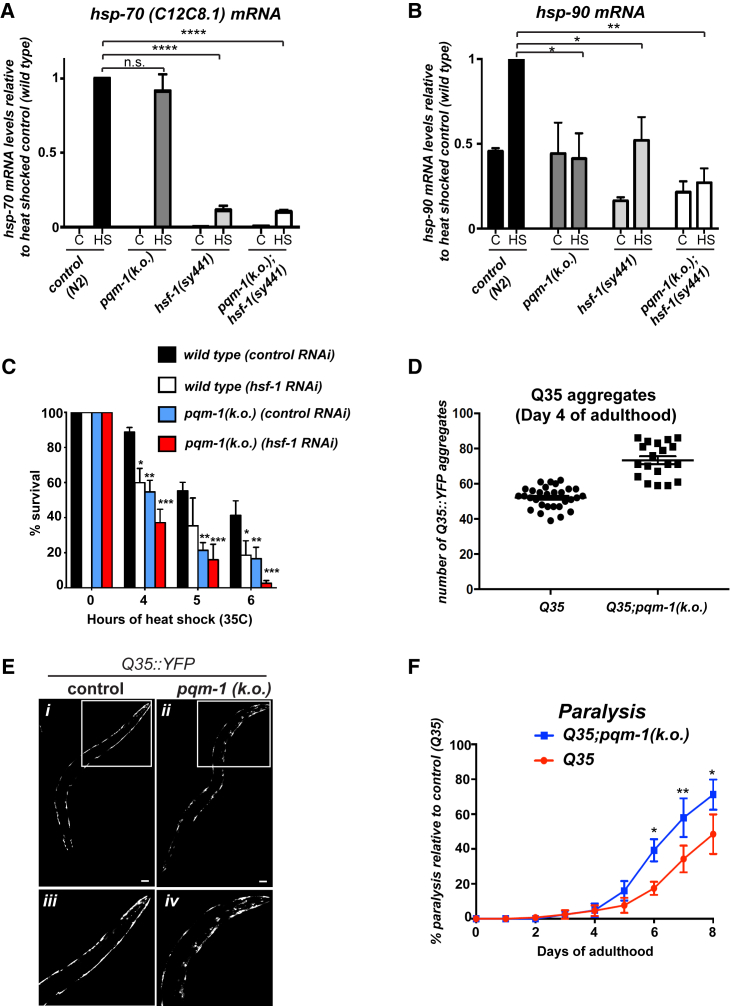
PQM-1 Regulates Heat-Inducible Expression of *hsp-90* and Is Involved in Proteostasis (A and B) mRNA levels of heat-inducible *hsp-70 (C12C8.1)* (A) or *hsp-90* (B) before and after heat shock (1 hr at 35°C) in young adult wild-type, *pqm-1(KO)* mutant, *hsf-1(sy441)* mutant, and *pqm-1(KO);hsf-1(sy441)* double-mutant animals relative to heat-shocked control animals (wild-type, N2). (C) Thermo-sensitivity of L4 animals (n = 20; five biological replicates) with indicated genotypes during control (EV) or *hsf-1* RNAi after exposure to a 35°C heat shock for 4, 5, or 6 hr. Survival was measured after a recovery period of 16 hr at 20°C. (D) *Q35::YFP* aggregation is enhanced in *pqm-1(KO)* mutants. Quantification of accumulated Q35 foci in age-synchronized day 4 adults of *pqm-1(KO)* mutants and control animals. ^∗^p < 0.05. (E) Day 4 adult *C. elegans* expressing *Q35::YFP* in the bodywall muscle of control animals (*i* and *iii*) and *pqm-1(KO)* mutants (*ii* and *iv*). 20x magnification of anterior (head) region of control and *pqm-1(KO)* mutants (*ii* and *iv*). Scale bar, 50 μm. (F) Q35-associated toxicity is increased in *pqm-1(KO)* mutants. Paralysis was measured in age-synchronized *pqm-1(KO)* mutants expressing *Q35::YFP* in the bodywall muscle (*Q35*) compared with *Q35* control animals at the indicated time points (100 animals per biological replicate, N = 3). ^∗^p < 0.05; ^∗∗^p < 0.01; paired t test. Error bars represent ± SEM. ^∗^p < 0.05; ^∗∗^p < 0.01; ^∗∗∗^p < 0.001, ^∗∗∗∗^p < 0.0001 (A–C). Bar graphs represent combined mean values of five independent experiments. Error bars represent ± SEM. See also Figures S3 and S4.

We measured mRNA levels of a range of heat-inducible chaperones, including stress-inducible *hsp-70 (C12C8.1*), *hsp-90,* the small HSP *hsp-16.2*, and constitutively expressed Hsp70 *(hsp-1)*, by qRT-PCR after a 1-hr heat shock (HS) at 35°C in *pqm-1(KO)* mutants (Figures 3A, 3B, S4C, and S4D). While induction of all four chaperones were significantly reduced in the *hsf-1(sy441)* mutant that cannot induce a proper HSR (Hajdu-Cronin et al., 2004), heat-inducible mRNA accumulation of *hsp-70, hsp-1*, and *hsp-16.2* was comparable between *pqm-1(KO)* and wild-type (WT) animals (Figures 3A, S4C, and S4D, respectively), as well as between the *hsf-1(sy441);pqm-1(KO)* mutant and *hsf-1(sy441)* (Figures 3A, S4C, and S4D), indicating that stress induction of these chaperones is dependent on functional *hsf-1*, but not *pqm-1*. Interestingly, both the *pqm-1(KO)* mutant and the *hsf-1(sy441);pqm-1(KO)* mutant showed significant decreases in heat-inducible *hsp-90* mRNA compared to controls (Figure 3B). After HS treatment, *hsp-90* was weakly induced in the *hsf-1(sy441)* mutant, albeit starting at a 50% lower basal expression level (Figure 3B). However, *hsp-90* mRNA induction after HS remained at its basal expression level in the *pqm-1(KO)* and *hsf-1(sy441);pqm-1(KO)* mutants (Figure 3B), with HSP-90 protein expression levels equally reduced in the *hsf-1(sy441)* and *pqm-1(KO)* compared to control animals (Figures S4E and S4F). This indicates that heat-inducible *hsp-90* expression is dependent on the presence of *pqm-1*, while basal *hsp-90* expression depends on functional *hsf-1*.

Next, we investigated whether loss of *pqm-1(KO)* has any consequences for HS survival of *C. elegans*. While 45% of control animals survived a 6-hr heat shock, only 20% of *pqm-1(KO)* mutants survived, comparable to animals treated with *hsf-1* RNAi (Figure 3C). RNAi-mediated knockdown of *hsf-1* in the *pqm-1(KO)* mutant further amplified this effect, with less than 5% of *pqm-1(KO)* animals treated with *hsf-1* RNAi surviving HS (Figure 3C). Similar results were obtained for *pqm-1(KO)* animals crossed into the genetic background of *hsf-1(sy441)* mutants (Figure S4G), indicating that *pqm-1* regulates heat stress survival in a pathway independent or complementary to *hsf-1*. These results show that although *pqm-1(KO)* mutants are capable of inducing *hsp-70* and *hsp-16.2* mRNA after HS, the inability of *pqm-1(KO)* mutants to induce protective levels of *hsp-90* is detrimental for survival.

### *pqm-1* Is Required for Proteostasis Maintenance

To assess whether *pqm-1* is required for the age-associated decline of proteostasis, we used *C. elegans* expressing Q35::YFP in the bodywall muscle (Morley et al., 2002). *Q35;pqm-1(KO)* animals accumulated an average of 70 aggregates per animal by day 4 of adulthood, whereas *Q35::YFP* control animals accumulated 55 aggregates (Figures 3D and 3E). At day 8 of adulthood, 75% of *Q35;pqm-1(KO)* animals were paralyzed compared to 50% of control animals, indicating increased toxicity (Figure 3F). Combined these results show that PQM-1 is a regulatory component of the proteostasis network that is required for stress survival and maintenance of proteostasis throughout aging.

### Nuclear Localization of PQM-1 Is Increased in the Intestine of *HSP-90::RFP*^*neuro*^

**Figure 4:**
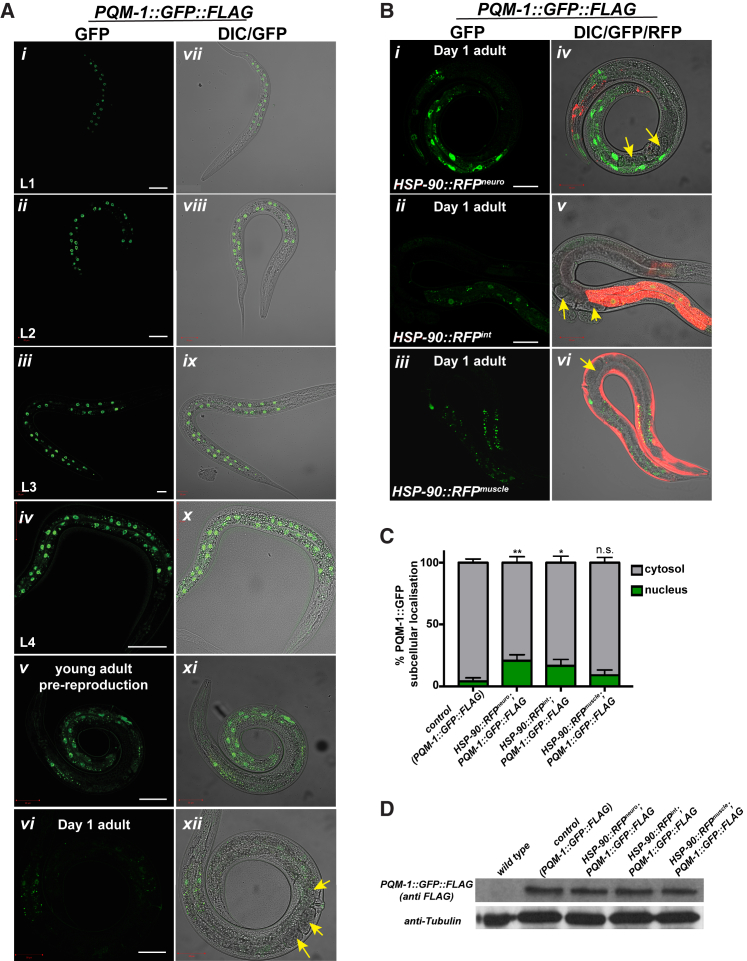
PQM-1::GFP::FLAG Remains Nuclearly Localized in the Intestine of Young Adult *C. elegans* Expressing *HSP-90::RFP*^*neuro*^ or *HSP-90::RFP*^*int*^ (A) Expression of PQM-1::GFP::FLAG (strain OP201) during larval stages L1(*i* and *vii*), L2 (*ii* and *viii*), L3 (*iii* and *ix*), and L4 (*iv* and *x*). PQM-1::GFP nuclear localization becomes diffuse in early pre-reproductive adults (56 hr after hatching at 20°C) (*v* and *xi*) and disappears in day 1 adults (65 hr after hatching) (*vi* and *xii*). Scale bar, 20 μm (*i–iii*). Scale bar, 50 μm (*iv-vi*). Yellow arrows denote eggs present in the day 1 adult. (B) PQM-1::GFP::FLAG localizes to intestinal nuclei in TCS-activated strains *HSP-90::RFP*^*neuro*^ (*i* and *iv*), *HSP-90::RFP*^*int*^ (*ii* and *v*), and *HSP-90::RFP*^*muscle*^ (*iii* and *vi*). Scale bar, 50 μm. Yellow arrows indicate eggs. (C) PQM-1::GFP::FLAG in *HSP-90::RFP*^*neuro*^, *HSP-90::RFP*^*int*^, or *HSP-90::RFP*^*muscle*^ was scored for nuclear and cytosolic localization and compared to the control strain (n > 20 per strain). ^∗^p < 0.05; ^∗∗^p < 0.01; Kruskal-Wallis test. Error bars represent ± SEM. (D) Western blot analysis of PQM-1::GFP::FLAG in TCS-activated strains compared to control animals using an anti-FLAG antibody. Tubulin was used as a loading control.

Our finding that PQM-1-dependent genes are upregulated in *HSP-90::RFP*^*neuro*^ in the neurons indicates that the transcriptional activity of PQM-1 must also be increased. Consistent with previous reports, we found PQM-1::GFP::FLAG protein nuclearly localized in intestinal cells throughout all larval stages (L1 to L4) in WT background, reflecting its active transcriptional role during development (Dowen et al., 2016, Tepper et al., 2013) (Figure 4A). In young pre-reproductive adults, nuclear localization of PQM-1 becomes more diffuse (Figures 4A*v* and 4A*xi*) before it completely disappears with the onset of egg production in day 1 adults (Figures 4A*vi* and 4*xii*). Interestingly, in TCS-activated adults *HSP-90::RFP*^*neuro*^ (Figures 4B*i* and 4B*iv*) or *HSP-90::RFP*^*int*^ (Figures 4B*ii* and 4B*v*) PQM-1 remained localized to the nucleus, signifying increased transcriptional activity, whereas no nuclear distribution of PQM-1 is observed in *HSP-90::RFP*^*muscle*^ (Figure 4B, *iii* and *vi*) or WT animals (Figures 4A*vi* and 4A*xii*). Quantification revealed that 25% of PQM-1::GFP::FLAG remained in intestinal nuclei in *HSP-90::RFP*^*neuro*^ and 20% in *HSP-90::RFP*^*int*^, compared to only 5% nuclear PQM-1 in the control strain and 10% (ns) in *HSP-90::RFP*^*muscle*^ (Figure 4C). However, PQM-1::GFP::FLAG protein levels remained constant across WT and TCS-activated strains (Figure 4D). Therefore, PQM-1 nuclear localization and presumably transcriptional activity is extended into reproductive adulthood through neuron- or intestine-specific overexpression of HSP-90.

Because *pqm-1* is required for resistance to the pathogenic bacteria *Pseudomonas aeruginosa* (Shapira et al., 2006), we asked whether the TCS-activated strains exhibiting increased nuclear PQM-1 localization were more resistant. Only *HSP-90::RFP*^*neuro*^ showed increased survival to *P. aeruginosa* (Figure S4H), consistent with the higher measured PQM-1 nuclear localization in this strain (Figures 4B and 4C). Depletion of *pqm-1* in *HSP-90::RFP*^*neuro*^, but not in control WT animals, reversed this effect (Figure S4H). Although PQM-1 nuclear localization is also induced in *HSP-90::RFP*^*int*^, it is lower when compared to *HSP-90::RFP*^*neuro*^ (Figures 4B and 4C) and may not be sufficient for an effective innate immune response.

### Neuron- and Intestine-Specific HSP-90 Overexpression Triggers PQM-1 Transcriptional Activity in the Same Tissue to Regulate Systemic TCS

PQM-1 is expressed in the intestine and neuronal cells (Reece-Hoyes et al., 2007, Tepper et al., 2013), although we could not detect PQM-1::GFP::FLAG expression by fluorescence microscopy in *C. elegans* neurons. We therefore wanted to determine whether PQM-1 is required in the neurons or the intestine of *HSP90::RFP*^*neuro*^ animals for TCS-mediated *hsp-90* induction in other tissues. Moreover, we considered the possibility that PQM-1 is activated in the bodywall muscle, where *P*_*hsp-90*_*::GFP* expression is largely induced as a consequence of TCS (Figures 2B–2D). We therefore generated *C. elegans* strains in which the effects of RNAi are restricted to neurons (*HSP-90::RFP*^*neuro*^;*P*_*hsp-90*_*::GFP, sid-1(pk3321);unc-119p::SID-1*) (Calixto et al., 2010), or intestine (*HSP-90::RFP*^*neuro*^;*P*_*hsp-90*_*::GFP, sid-1(pk3321);vha-6p::SID-1)*, or bodywall muscle (*HSP-90::RFP*^*neuro*^;*P*_*hsp-90*_*::GFP, sid-1(pk3321);unc-54p::SID-1).*

To confirm that tissue-specific RNAi was indeed restricted to neurons, intestine, or muscle, we exposed the aforementioned *C. elegans* strains to *hsp-90* RNAi and measured tissue-specific HSP-90::RFP fluorescence intensity (Figure S5). For example, HSP-90::RFP fluorescence intensity in the neurons was reduced to 25% by systemic *hsp-90* RNAi (Figures S5A*a* and S5A*b*) and to 30% by neuron-specific *hsp-90* RNAi (Figures S5C*a* and S5C*b*), but had no effect upon intestine-specific or muscle-specific *hsp-90* RNAi (Figures S5D*a* and S5D*b* and Figures S5E*a* and S5E*b*, respectively). Consistently, *hsp-90* RNAi in the RNAi-resistant control strain *HSP-90::RFP*^*neuro*^;*P*_*hsp-90*_*::GFP;sid-1(pk3321)* did not reduce HSP-90::RFP expression (Figures S5B*a* and S5B*b*). Similar results showing the tissue specificity of this RNAi approach were also obtained in *HSP-90::RFP*^*int*^ and *HSP-90::RFP*^*muscle*^ strains (see Figure S5).

**Figure 5:**
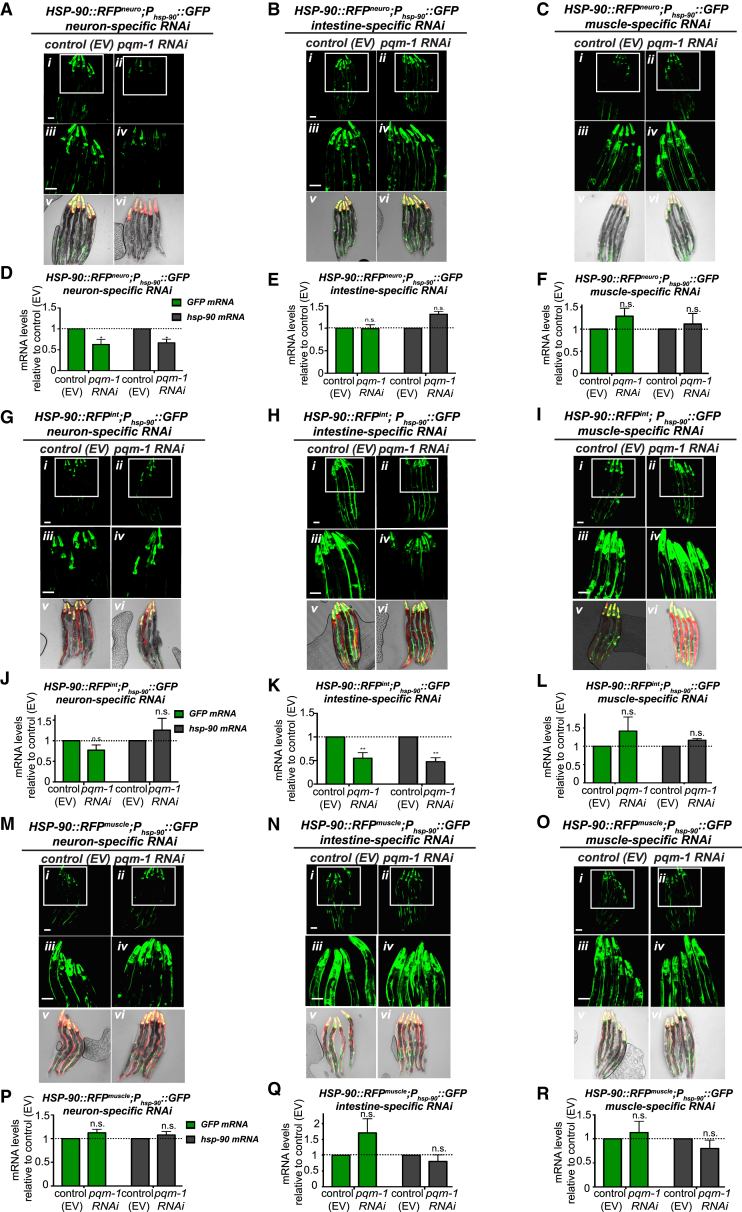
PQM-1 Is Required in the Neurons in *HSP-90::RFP*^*neuro*^ and in the Intestine in *HSP-90::RFP*^*int*^ to Induce TCS (A–C) Expression of the *P_hsp-90_::GFP* transcriptional reporter in *HSP-90::RFP^neuro^* during tissue-specific control or *pqm-1* RNAi in neurons (A), intestine (B), or bodywall muscle (C). (D–F) Transcript levels of *GFP* and *hsp-90* in *HSP-90::RFP^neuro^;P_hsp-90_::GFP* during neuron-specific (D), intestine-specific (E), and muscle-specific (F) control (EV) or *pqm-1* RNAi. ^∗^p < 0.05; ns, non-significant. Bar graphs represent combined mean values of three independent experiments. Error bars represent ± SEM. (G–I) Expression of the *P_hsp-90_::GFP* transcriptional reporter in *HSP-90::RFP^int^* adults during tissue-specific control RNAi (EV) or *pqm-1* RNAi in neurons (G), intestine (H), or bodywall muscle (I). (J–L) Transcript levels of *GFP* and *hsp-90* during neuron-specific (J), intestine-specific (K), or muscle-specific (L) control (EV) or *pqm-1* RNAi in *HSP-90::RFP^int^;P_hsp-90_::GFP*. (M–O) Expression of the *P_hsp-90_::GFP* transcriptional reporter in *HSP-90::RFP^muscle^* adults during tissue-specific control RNAi (EV) or *pqm-1* RNAi in neurons (M), intestine (N), or bodywall muscle (O). (P–R) Transcript levels of *GFP* and *hsp-90* mRNA in *HSP-90::RFP^muscle^;P_hsp-90_::GFP* during neuron-specific (P), intestine-specific (O), or muscle-specific (R) control (EV) or *pqm-1* RNAi. ^∗^p < 0.05; ns, non-significant. Bar graphs represent combined mean values of three independent experiments. Error bars represent ± SEM (A–C, G–I, and M–O). Scale bar, 50 μm. See also Figures S5 and S6.

Neuron-specific *pqm-1* RNAi in *HSP-90::RFP*^*neuro*^ effectively reduced GFP fluorescence intensity of the *P*_*hsp-90*_*::GFP* reporter compared to animals treated with control RNAi (Figure 5A), corresponding to a 40% reduction of both *GFP* and *hsp-90* mRNA levels (Figure 5D). Interestingly, intestine-specific knockdown of *pqm-1* by RNAi in *HSP-90::RFP*^*neuro*^ had no effect on *P*_*hsp-90*_*::GFP* reporter expression (Figure 5B) or *GFP* and *hsp-90* mRNA levels (Figure 5E). These results suggest that PQM-1 is required in the neurons, but not the intestine, of *HSP-90::RFP*^*neuro*^ to mediate TCS-induced *hsp-90* expression in distal tissues. The higher distribution of PQM-1 to intestinal nuclei in this strain (Figures 4B and 4C) may be a consequence of TCS but is not required for TCS-mediated *hsp-90* expression.

Intestine-specific *pqm-1* RNAi in *HSP-90::RFP*^*int*^ reduced GFP fluorescence intensity of the *P*_*hsp-90*_*::GFP* reporter (Figure 5H) which corresponded to a 50% decrease of *GFP* and *hsp-90* mRNA expression relative to control animals (Figure 5K). However, neuron-specific *pqm-1* RNAi in the same strain left *P*_*hsp-90*_*::GFP* reporter expression unchanged (Figure 5G) and had no effect on *GFP* or *hsp-90* mRNA expression levels (Figure 5J). Combined, these results indicate that PQM-1 may be activated in the same tissue that overexpresses HSP-90::RFP to induce TCS, but does not require PQM-1 to be expressed in other tissues for TCS. Interestingly, muscle-specific *pqm-1* RNAi had no effect on TCS-mediated *hsp-90* expression in *HSP-90::RFP*^*neuro*^ (Figures 5C and 5F), *HSP-90::RFP*^*int*^ (Figures 5I and 5L) or *HSP-90::RFP*^*muscle*^ (Figures 5O and 5R). This indicates that neurons and intestine are key tissues for TCS and suggests that PQM-1 indirectly regulates *hsp-90* expression in the muscle.

### PQM-1 Indirectly Regulates TCS-Induced *hsp-90* Expression in Muscle via Innate Immune Factors *clec-41* and *asp-12*

Next, we investigated whether PQM-1 directly regulates TCS-induced *hsp-90* expression by binding to a putative PQM-1 binding motif 119–129 bp upstream of the TSS in the *hsp-90* promoter. Therefore we generated a transcriptional reporter containing the native *hsp-90* promoter region upstream of GFP, *P*_*hsp-90*_*(WT)::GFP,* or lacking the putative PQM-1 binding motif sequence, *P*_*hsp-90*_*(del)::GFP* (Figure S6A). *P*_*hsp-90*_*(del)::GFP* reporter animals did however not show a significant change in GFP intensity relative to the native *P*_*hsp-90*_*(WT)::GFP* reporter (Figures S6B and S6D). Although both the native or modified *P*_*hsp-90*_*::GFP* reporter exhibited a significant upregulation of *P*_*hsp-90*_*::GFP* expression in the bodywall muscle and pharyngeal tissues when crossed into *HSP-90::RFP*^*neuro*^ (Figure S6C), this TCS-mediated *hsp-90* induction was similar between the native and modified reporter constructs (Figures S6C, S6D, and S6E). Thus, deletion of the putative PQM-1 binding motif in the *hsp-90* promoter had no effect on TCS-mediated *hsp-90* expression in distal tissues, corroborating the idea that PQM-1 indirectly controls *hsp-90* expression possibly via a *pqm-1*-regulated effector.

**Figure 6:**
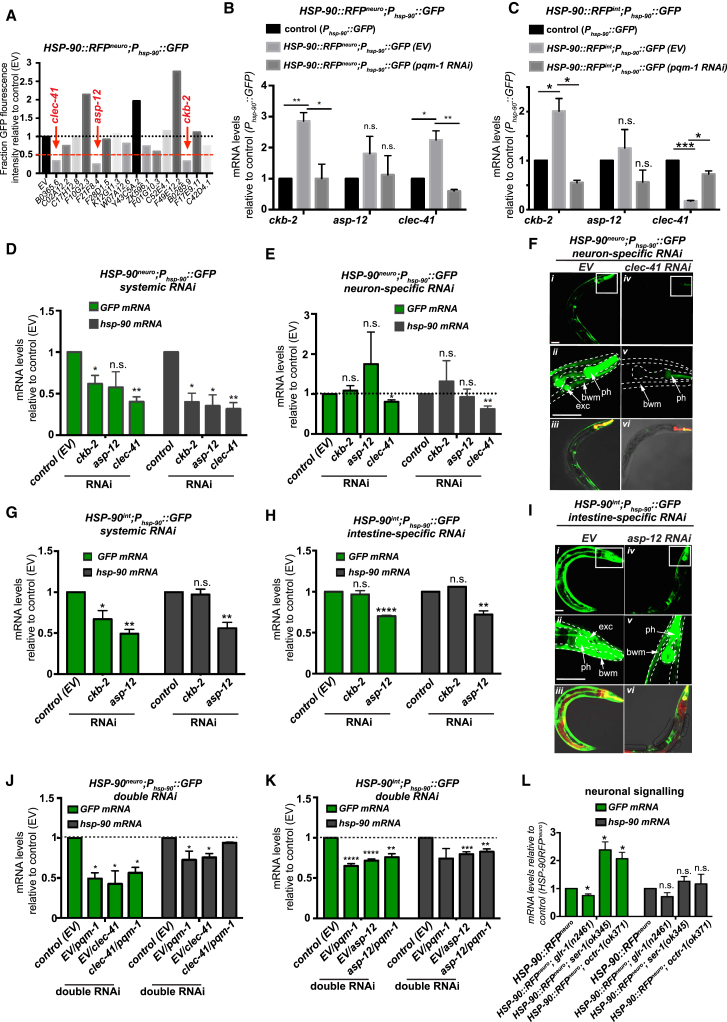
*pqm-1*-Regulated Genes Are Required for TCS-Induced *hsp-90* Expression (A) Quantification of *P*_*hsp-90*_*::GFP* fluorescence intensity in *HSP-90::RFP*^*neuro*^;*P*_*hsp-90::*_*GFP* animals during RNAi-mediated knockdown of indicated 16 genes relative to control (EV) RNAi. Scored hits (*clec-41, asp-12,* and *ckb-2*) show less than 50% GFP fluorescence intensity. (B) mRNA levels of *ckb-2 (B0285.9)* and innate immune factors *asp-12 (F21F8.4)* and *clec-41 (B0354.5)* in *HSP-90*^*neuro*^*;P*_*hsp*_*-90::GFP* animals treated with control or *pqm-1* RNAi. (C) Total mRNA levels of *pqm-1*-regulated kinase *ckb-2 (B0285.9)* and innate immune factors *asp-12 (F21F8.4)* and *clec-41 (B0354.5)* in *HSP-90*^*int*^*;P*_*hsp*_*-90::GFP* animals treated with control or *pqm-1* RNAi. (D) Whole-animal (systemic) RNAi knockdown of *ckb-2, asp-12*, or *clec-41* in *HSP-90*^*neuro*^*;P*_*hsp*_*-90::GFP* reduces transcript levels of GFP and *hsp-90*. (E) Neuron-specific RNAi of *clec-41*, but not *ckb-2* or *asp-12*, reduces GFP and *hsp-90* mRNA levels in *HSP-90*^*neuro*^*;P*_*hsp*_*-90::GFP;sid-1(pk3321);unc-119p::SID-1*. (F) Collapsed z stack images of *HSP-90::RFP*^*neuro*^*;P*_*hsp-90*_*::GFP;sid-1(pk3321);unc-119p::SID-1* animals allowing for neuron-specific EV control RNAi (*i*) or *clec-41* RNAi (*iv*). 20x magnification of the anterior (head) region (*ii* and *v*). Differential interference contrast (DIC) Nomarski GFP, RFP overlay images (*iii* and *vi*). (G) Whole-animal (systemic) RNAi knockdown of *ckb-2* and *asp-12* in *HSP-90*^*int*^*;P*_*hsp*_*-90::GFP* reduces GFP and *hsp-90* mRNA levels. (H) Intestine-specific RNAi of *asp-12*, but not *ckb-2*, reduces GFP and *hsp-90* mRNA levels in *HSP-90*^*int*^*;P*_*hsp*_*-90::GFP sid-1(pk3321);vha-6p::SID-1*. (I) Collapsed z stack images of *HSP-90::RFP*^*int*^*;P*_*hsp-90*_*::GFP;sid-1(pk3321);vha-6p::SID-1* animals allowing for intestine-specific control RNAi (*i*) or *asp-12* RNAi (*iv*). 20x magnification of the anterior (head) region (*ii* and *v*). Differential interference contrast (DIC) Nomarski images (*iii* and *vi*). (J) Simultaneous *pqm-1* and *clec-41* RNAi (1:1 ratio) does not further reduce *GFP* mRNA and *hsp-90* mRNA levels in *HSP-90*^*neuro*^*;P*_*hsp*_*-90::GFP*. (K) Simultaneous *pqm-1* and *asp-12* RNAi (1:1 ratio) does not further reduce *GFP* mRNA and *hsp-90* mRNA levels in *HSP-90*^*int*^*;P*_*hsp*_*-90::GFP.* (L) *GFP* and *hsp-90* transcript levels in *HSP-90::RFP*^*neuro*^*; P*_*hsp-90*_*::GFP* animals crossed into the genetic background of *glr-1(n2461)*, *ser-1(ok345)*, or *octr-1(ok371)* mutants relative to control animals. Bar graphs represent combined mean values of three independent experiments (B–E, G, H, J, K, and L). Error bars represent ± SEM. ^∗∗∗∗^p < 0.0001; ^∗∗^p < 0.01; ^∗^p < 0.05; ns, not significant. See also Figure S7.

To examine this possibility further, we performed an RNAi mini-screen of the 16 genes identified in our RNA-seq dataset that contain a PQM-1 binding motif in their promoters (Niu et al., 2011) (Table S2). We identified three hits, namely *F21F8.4 (asp-12)*, *B0365.6 (clec-41)*, and *B0285.9 (ckb-2)* that upon RNAi-mediated knockdown reduced GFP fluorescence by more than 50% in *HSP-90::RFP*^*neuro*^;*P_hsp-90_::GFP* (Figure 6A and Figure S7A). mRNA expression of all three genes are induced in *HSP-90::RFP*^*neuro*^;*P*_*hsp-90*_*::GFP* (Figure 6B) and RNAi-mediated knockdown of *pqm-1* reduced mRNA expression of all three genes back to control levels (Figure 6B), confirming the requirement for *pqm-1* to regulate their expression. Systemic *ckb-2, asp-12*, or *clec-41* RNAi in *HSP-90::RFP*^*neuro*^ reduced *P*_*hsp-90*_*::GFP* (Figure S7A), as well as *GFP* and *hsp-90* mRNA expression relative to control RNAi, indicating their involvement in TCS-mediated *hsp-90* induction (Figure 6D).

### PQM-1 Orchestrates Neuron-Activated TCS via PQM-1/CLEC-41 and Intestine-Activated TCS via PQM-1/ASP-12 Signaling

*HSP-90::RFP*^*neuro*^ requires PQM-1 in the neurons to trigger TCS; therefore, we tested whether neuronal expression of *ckb-2, asp-12*, or *clec-41* is required in the neurons for TCS-mediated *hsp-90* induction in the muscle. Strikingly, neuron-specific RNAi of only one of the gene hits, *clec-41*, reduced TCS-induced *hsp-90* expression (Figure 6E). At the level of individual tissues this corresponded to reduced expression of the *P*_*hsp-90*_*::GFP* reporter in bodywall muscle and pharynx, indicating the requirement for *clec-41* expression in the neurons to mediate TCS-induced expression of *hsp-90* in muscle (Figures 6F and S7C). Simultaneous *clec-41* and *pqm-1* RNAi did not lead to further reduction of TCS activity (Figure 6J), suggesting that *pqm-1* and *clec-41* function in the same signaling pathway to regulate TCS-mediated *hsp-90* induction.

Because *HSP-90::RFP*^*int*^ induces TCS in a *pqm-1* dependent manner, we sought to determine whether *ckb-2, asp-12* or *clec-41* could also play a role in TCS-mediated *hsp-90* expression in *HSP-90::RFP*^*int*^. Indeed, both *ckb-2* and *asp-12* expression were upregulated (Figure 6C). Systemic *ckb-2* or *asp-12* RNAi significantly reduced *GFP* mRNA expression in *HSP-90::RFP*^*int*^;*P*_*hsp-90*_*::GFP*, with *asp-12* RNAi leading to a significant 50% decrease of global *hsp-90* mRNA expression relative to control RNAi (Figures 6G and S7D). Intestine-specific *ckb-2* RNAi was ineffective, but intestine-specific *asp-12* RNAi reduced global *hsp-90* levels to 75% (Figure 6H), and reduced *P*_*hsp-90*_*::GFP* reporter expression in muscle cells (Figures 6I and S7E). Simultaneous *asp-12* and *pqm-1* RNAi led to no further reduction of *hsp-90* or *GFP* mRNA expression levels, confirming that *asp-12* and *pqm-1* likely function in the same intestine-activated TCS pathway (Figure 6K). Importantly, the neuron-induced TCS route was unaffected via intestine-specific *clec-41* or *asp-12* RNAi (Figure S7I), whereas intestine-activated TCS was unaffected by neuron-specific *clec-41* or *asp-12* RNAi (Figure S7J), demonstrating that both TCS routes function independent of each other and are differentially regulated.

### Neuron-Induced TCS Promotes Non-cell-autonomous *hsp-90* Expression through Glutamate-Mediated Neurotransmission

We have previously shown that TCS-mediated *hsp-90* expression is controlled independently of dense-core vesicle *unc-31-* or small core vesicle *unc-13-* mediated neuro-secretion (van Oosten-Hawle et al., 2013), yet neuron-induced TCS regulates *hsp-90* expression in distal tissues via neuronally activated PQM-1/CLEC-41 signaling. One explanation for this discrepancy is that upon depletion of *unc-31*-mediated dense core vesicle neuro-secretion the *unc-13*-mediated small core vesicle route is still active and so compensates for loss of the other and vice versa. Depletion of *unc-31* had no effect on TCS-mediated *hsp-90* or *GFP* mRNA expression (Figures S7F–S7H) (van Oosten-Hawle et al., 2013), and additional RNAi-mediated knockdown of *unc-13* in the neurons of these strains did not alter global *hsp-90* or *GFP* mRNA expression (Figures S7F–S7H). Nevertheless, a signal needs to be transmitted from neurons to distal tissues in *HSP-90::RFP*^*neuro*^. We therefore tested neurotransmitter-mediated pathways previously identified to be required for systemic stress signaling in *C. elegans*, such as serotonergic and octopaminergic signaling (Sun et al., 2012, Tatum et al., 2015b) for their involvement in TCS. *HSP-90::RFP*^*neuro*^ animals deficient for serotonergic signaling via deletion of the metabotropic serotonin receptor *ser-1*, exhibited increased global *GFP* and *hsp-90* mRNA expression (Figure 6L), similar to *HSP-90::RFP*^*neuro*^ animals deficient for octopaminergic signaling, via depletion of the G-protein-coupled receptor *octr-1* (Figure 6L). Thus, serotonergic and octopaminergic signaling are both involved in TCS; however, they may function as suppressors rather than facilitators.

The innate immune peptide CLEC-41 is a predicted plasma membrane-associated protein containing two beta-barrel forming domains known as “CUB domains,” which are found almost exclusively in extracellular and plasma membrane-associated proteins (Bork and Beckmann, 1993) and enriched in the neurons and intestine (Reece-Hoyes et al., 2007, Spencer et al., 2011). Interestingly, other neuronally expressed CUB domain proteins in *C. elegans*, e.g., *sol-1*, are known to participate in the gating mechanism for ions to pass through AMPA-type glutamate receptors (Zheng et al., 2006). We therefore investigated the role of glutamatergic signaling for TCS and used a *glr-1* mutant that expresses a non-functional version of the ionotropic AMPA-type glutamate receptor (Rongo et al., 1998) in *HSP-90::RFP*^*neuro*^. This decreased *GFP* and global *hsp-90* mRNA expression by 25% relative to the control (Figure 6L), suggesting that glutamatergic signaling is indeed a neuronal signaling route required for TCS.

### TCS via Neuronal PQM-1/CLEC-41 Signaling and Intestinal PQM-1/ASP-12 Signaling Protects against *mAβ*-Associated Toxicity

**Figure 7:**
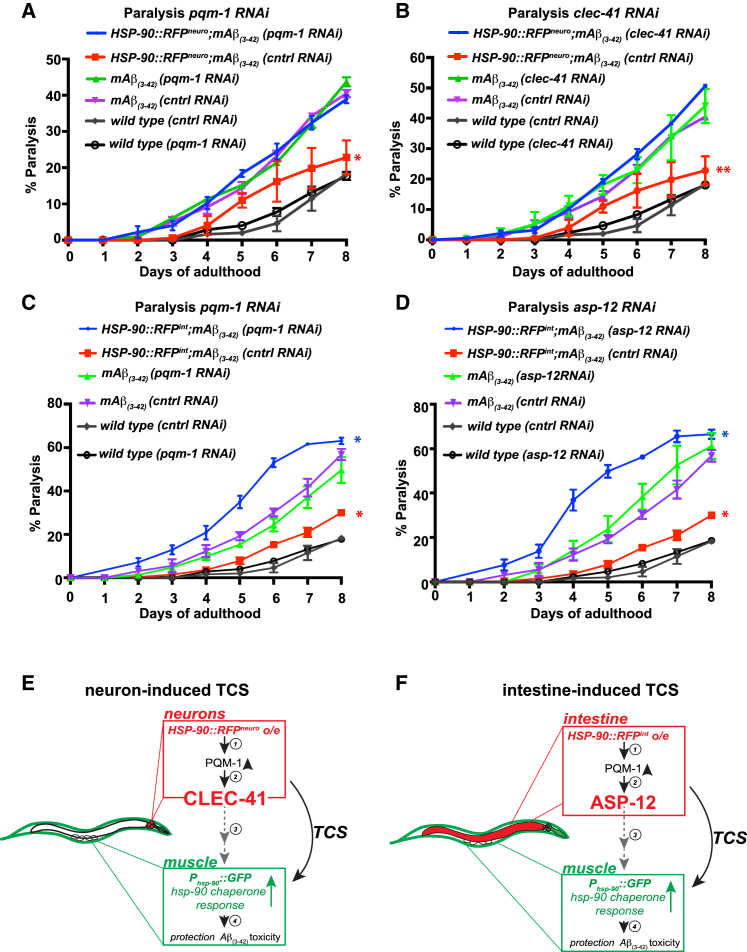
The *pqm-1*-Regulated Immune Factors *clec-41* and *asp-12* Are Required for Neuron- and Intestine-Induced TCS, Respectively, to Protect against *Aβ*_*(3-42)*_ Toxicity in the Muscle (A) *pqm-1* RNAi exacerbates paralysis of *HSP-90::RFP*^*neuro*^*;Aβ*_*(3-42)*_ compared with control (EV) RNAi. (B) *clec-41* RNAi exacerbates paralysis of *Aβ*_*(3-42)*_ animals overexpressing HSP-90 in the neurons (*HSP-90::RFP*^*neuro*^*;Aβ*_*(3-42)*_). (C) *pqm-1* RNAi exacerbates paralysis of *HSP-90::RFP*^*int*^*;Aβ*_*(3-42)*_. (D) *asp-12* RNAi exacerbates paralysis of Aβ_(3-42)_ animals overexpressing HSP-90 in the intestine (*HSP-90::RFP*^*int*^*;Aβ*_*(3-42)*_). (A–D) Paralysis data error bars represent SEM of 3 biological replicates (>100 animals per replicate). Statistical significance was determined by Wilcoxon matched-pairs signed-rank test, comparing *HSP-90::RFP*^*neuro*^*;Aβ*_*(3-42)*_ grown on *pqm-1 or clec-41* RNAi or *HSP-90::RFP*^*int*^*;Aβ*_*(3-42)*_ grown on *pqm-1* or *asp-12* RNAi with control (EV) RNAi. ^∗^p < 0.05, ^∗∗^p < 0.01. (E) Model explaining neuron-induced TCS activation of *hsp-90* chaperone expression in the muscle via PQM-1/CLEC-41 signaling. (1) Neuronal overexpression of HSP-90::RFP induces PQM-1 activity locally. (2) Increased PQM-1 activity in the neurons induces transcription of *clec-41*, which aids in the transmission of a “transcellular chaperone signal” (3) that activates *hsp-90* expression in muscle cells (4) to protect them from Aβ_(3-42)_-associated toxicity. (F) Model explaining intestine-induced TCS activation of *hsp-90* in the muscle via PQM-1/ASP-12 signaling. (1) Intestine-specific overexpression of HSP-90::RFP induces PQM-1 activity cell autonomously in the intestine. (2) Active PQM-1 in the intestine induces transcription of *asp-12*, which is (3) required to induce *hsp-90* expression in muscle cells (4) to protect them from Aβ_(3-42)_-associated toxicity.

Neuron-induced and intestine-induced TCS mediate *hsp-90* induction in the muscle, thereby alleviating muscle-expressed *mAβ*_*3-42*_ -associated toxicity (Figure 1E). Remarkably, *pqm-1* or *clec-41* RNAi in the TCS-activated *HSP-90::RFP*^*neuro*^*;mAβ*_*3-42*_ strain exacerbated paralysis (Figures 7A and 7B, respectively) reversing the protective effect of neuron-induced TCS. While 23% of *HSP-90::RFP*^*neuro*^*;mAβ*_*3-42*_ animals grown on control RNAi were paralyzed at day 8 of adulthood, *pqm-1* RNAi in *HSP-90::RFP*^*neuro*^*;mAβ*_*3-42*_ abolished the TCS-mediated protective effect by increasing the amount of paralysed *HSP-90::RFP*^*neuro*^*;mAβ*_*3-42*_ animals to 38% (Figure 7A), comparable to *mAβ*_*3-42*_ control animals (Figure 7A). Likewise, *pha-4* RNAi reversed the protective effect of *HSP-90::RFP*^*neuro*^ on amyloid toxicity in the bodywall muscle (Figure S7K). *Clec-41* RNAi resulted in 50% of *HSP-90::RFP*^*neuro*^*;mAβ*_*3-42*_ animals being paralyzed at day 8 (Figure 7B), reducing the TCS-mediated protection against amyloid toxicity. However, *pqm-1* or *clec-41* RNAi in *mAβ*_*3-42*_ control animals alone did not further increase the fraction of paralysed *mAβ*_*3-42*_ animals (Figures 7A and 7B), indicating that the protective effect via PQM-1/CLEC-41 signaling was specific to neuronal activation of TCS. Strikingly, *pqm-1* and *asp-12* RNAi during intestine-induced TCS led to an even more severely exacerbated paralysis of *HSP-90::RFP*^*int*^*;mAβ*_*3-42*_ compared to the *mAβ*_*3-42*_ control strain (Figures 7C and 7D, respectively). In summary, our data show that neuron-induced TCS mediates suppression of amyloid protein-associated toxicity via *hsp-90* induction in the muscle dependent on neuronal PQM-1/CLEC-41 signaling, while the same effect via intestine-induced TCS is dependent on PQM-1/ASP-12 signaling.

## Discussion

Our study identified key components of signaling cues that regulate transcellular activation of *hsp-90* chaperone expression in *C. elegans*. We identified PQM-1 as a mediator of TCS that differentially orchestrates the induction of innate immune peptides to promote *hsp-90* expression in distal tissues. Activation of TCS in the neurons requires the plasma-membrane-associated innate immune peptide CLEC-41 in the neurons (Figure 7E), while activation of TCS in the intestine depends on the aspartic protease ASP-12 to induce *hsp-90* expression in distal tissues (Figure 7F). Both TCS cues—the neuron-activated PQM-1/CLEC-41 signaling cue and the intestine-activated PQM-1/ASP-12 signaling pathway—induce *hsp-90* in muscle cells to ameliorate *Aβ*_*3-42*_ -associated toxicity and reduce the accumulation of toxic oligomeric amyloid species.

The PQM-1-dependent TCS network appears to require PQM-1 activity in the stressed sender tissue and not the responding tissue (Figures 7E and 7F). While *hsp-90* is ubiquitously expressed in *C. elegans* (Casanueva et al., 2012, Mendenhall et al., 2015, van Oosten-Hawle et al., 2013), expression of *pqm-1* overlaps with *hsp-90* only in neuronal and intestinal tissue (Reece-Hoyes et al., 2007, Spencer et al., 2011). Moreover, *pqm-1* does not appear to be required in the muscle to directly regulate *hsp-90* expression. Further evidence arguing against the possibility of PQM-1 directly regulating *hsp-90* expression, is provided via deletion of the putative PQM-1 GATA binding site in the *hsp-90* promoter. However, before completely excluding this possibility, a more detailed promoter analysis that includes >1000 bp of the *hsp-90* promoter or ChIP-seq experiments will be needed to characterize PQM-1’s role in the induction of stress-induced *hsp-90* expression. To this end, our data suggest that PQM-1 regulates *hsp-90* expression via an indirect mechanism that involves *pqm-1* regulated innate immune genes *asp-12* and *clec-41*.

While neither ASP-12 nor CLEC-41 act as the “transcellular chaperone activating signal” itself, as neither are predicted secreted peptides (Table S2), both could facilitate the transmission of a “signal” to other tissues. As a CUB domain containing protein expressed in neurons (Zheng et al., 1999), CLEC-41 could be a factor that facilitates neuron-activated TCS via glutamatergic signaling. Future work should define the neuron-specific function for CLEC-41 in TCS in more detail.

Many known PQM-1 target genes are associated with the innate immune response (Niu et al., 2011, Shapira et al., 2006, Templeman et al., 2018) and are also identified in our TCS-induced dataset. While our results indicate a clear requirement for *pqm-1* for *P. aeruginosa* resistance during neuronal activation of TCS, this is not the case in the *pqm-1(KO)* mutant alone (Figure S4H) and contrary to a previous report (Shapira et al., 2006). This discrepancy may however stem from differences in strain backgrounds used (*pqm-1(KO)* versus *pqm-1* RNAi in a *glp-4 (bn2);rrf-3* mutant) and different assay temperatures (20°C vs. 25°C). It may be that an increased temperature (25°C) poses an additional challenge that affects *pqm-1*-dependent survival on pathogenic bacteria.

Our discovery of PQM-1 as an important component of the PN reveals that metazoans make use of non-canonical stress transcription factors that function complementary to HSF-1 and DAF-16 to control proteostasis. For example, HSP-90 chaperone expression can be regulated by the myogenic transcription factor HLH-1 during *C. elegans* muscle development (Bar-Lavan et al., 2016) and by PHA-4 during TCS (van Oosten-Hawle et al., 2013).

Moreover, *C. elegans* utilizes multiple regulatory pathways to specify the type of stress-response activated in remote cells. For example, DAF-16-mediated stress-resistance is separated from HSF-1-mediated activity through a distinct chemosensory neuronal circuit (Volovik et al., 2014), whereas HSF-1 uses a thermo-sensory circuit to promote non-cell-autonomous expression of HSPs (Douglas et al., 2015, Prahlad and Morimoto, 2011, Prahlad et al., 2008). Likewise, activation of UPR pathways in response to bacterial infection utilizes octopaminergic signaling (Sun et al., 2012).

Similar to the differential regulatory cues initiated via HSF-1 or DAF-16, stress conditions that activate TCS also utilize more than one regulatory mechanism. TCS-mediated *hsp-90* expression via PHA-4 is regulated differently from the PQM-1-dependent signaling routes described here. Moreover, neuronal activation of TCS triggers a PQM-1-dependent signaling route that relies on CLEC-41 and glutamatergic signaling, whereas activation of TCS from the intestine requires ASP-12 as a key transcellular signaling node.

In neuron-activated TCS, global *hsp-90* expression is induced 2.5-fold, whereas *hsp-90* expression is induced 1.75-fold in *HSP-90::RFP*^*int*^, which is lower compared to our previous study using the same strain (van Oosten-Hawle et al., 2013). It is possible that this measured discrepancy is due to suppressor mutations that have accumulated in *HSP-90::RFP*^*int*^. Despite this difference in the level of *hsp-90* induction, both *HSP-90::RFP*^*int*^ and *HSP-90::RFP*^*neuro*^ induce TCS, which results in a substantial and similar suppression of the toxic effects of Aβ expression in the muscle in both strains (Figures 1E–1G), comparable to the previously observed suppression of metastable myosin misfolding in muscle (van Oosten-Hawle et al., 2013). Although monomer and smaller oligomeric Aβ species (< 17 kDa) are unaffected by TCS-induced *hsp-90* expression in *HSP-90::RFP*^*neuro*^ and *HSP-90::RFP*^*int*^, both significantly reduce the accumulation of higher Aβ oligomers (>26 kDa). Thus HSP-90 may be involved in the assembly of Aβ oligomers, as has been suggested *in vitro* (Evans et al., 2006), and this may be comparable to the suppressive effect of HSP-16.2 overexpression in the Aβ_3-42_ *C. elegans* model (Fonte et al., 2008). In this light, it is also interesting to note that increased expression of the HSP90 co-chaperone STI1 protects against the toxic effects of Aβ expression in neuronal cells and a mouse model of Alzheimer’s disease (Ostapchenko et al., 2013). Further work would be required to understand how HSP-90 reduces formation of Aβ oligomers *in vitro* and *in vivo*. Moreover, it would be interesting to determine whether modulation of the neuronal PQM-1/CLEC-41 or the intestinal PQM-1/ASP-12 pathway, via genetic activation or small molecules could provide an avenue for the treatment of protein misfolding diseases.

## Experimental Procedures

### *C. elegans* Growth Conditions

*C. elegans* were grown on nematode growth media (NGM) plates seeded with the *E. coli OP50-1* strain and cultured by standard methods (Brenner, 1974). Strains were obtained from the *Caenorhabditis* Genetic Center (CGC). For a detailed description of *C. elegans* strains generated in this study, please refer to Supplemental Experimental Procedures.

### RNAi Experiments

For RNAi-mediated knockdown of indicated genes, 50 synchronized L1 animals were placed on *E. coli* strain HT115(DE3), which was transformed with the appropriate RNAi vector (J. Ahringer, University of Cambridge, Cambridge, UK), as described previously (van Oosten-Hawle et al., 2013). For RNAi-mediated knockdown of *hsp-90* or *hsf-1*, L4 stage larvae instead of L1 larvae were placed on the appropriate RNAi.

### qRT-PCR

RNA was extracted using TriZOL reagent and grinding *C. elegans* using a pellet grinder on ice 3× for 30 s. RNA was then purified using the Zymo-prep RNA Mini Isolation kit (Zymo Research, Cambridge Biosciences). qRT-PCR was essentially performed as described previously (van Oosten-Hawle et al., 2013).

### Statistical Analysis

All experiments were repeated at least three times (3 biological replicates). Data are presented as mean values ± SEM. p values were calculated using Student’s t test. ^∗^ denotes p < 0.05; ^∗∗^ denotes p < 0.01, and ^∗∗∗^ denotes p < 0.001; (ns) denotes non-significant. To compare two independent populations (paralysis assays), we calculated p values using the Wilcoxon Mann-Whitney rank-sum test. Prism (v.7) software was used for all statistical analysis.

Detailed protocols for all the methods are provided in the Supplemental Experimental Procedures.

## Data and Software Availability

The accession number for the full RNA-seq dataset reported in this study is GEO: GSE108005.

## Supplemental Information

Document S1. Supplemental Experimental Procedures and Figures S1–S7

Table S1. Differentially Expressed Genes in C. elegans Overexpressing HSP-90::GFP in the Neurons (HSP-90::GFPneuro) Relative to Control Animals (N2) at Normal Growth Conditions (20°C), Related to Figures 2 and S1

Table S2. Genes Upregulated >1.5-Fold in C. elegans Overexpressing HSP-90::GFP in the Neurons (HSP-90::GFPneuro), Related to Figures 2 and S1 Pseudogenes are indicated in red. Y = Yes; N = No

Document S2. Article plus Supplemental Information
