# Supplementary material for: A PQM-1-Mediated Response Triggers Transcellular Chaperone Signaling and Regulates Organismal Proteostasis
Source: Cell Rep. 2018 Jun 26;23(13):3905–19. doi: 10.1016/j.celrep.2018.05.093 (PMC6045774; doi:10.1016/j.celrep.2018.05.093)
Supplement: Document S1. Supplemental Experimental Procedures and Figures S1–S7 [file mmc1.pdf]

Cell Reports, Volume *23*

Supplemental Information

A PQM-1-Mediated Response Triggers Transcellular Chaperone Signaling and Regulates Organismal Proteostasis

Daniel O'Brien, Laura M. Jones, Sarah Good, Jo Miles, M.S. Vijayabaskar, Rebecca Aston, Catrin E. Smith, David R. Westhead, and Patricija van Oosten-Hawle

SUPPLEMENTAL INFORMATION

SUPPLEMENTAL EXPERIMENTAL PROCEDURES

Nematode strains

Strains RB711 (*pqm-1(ok435)*), OP201(*wgIs201 [pqm-1::TY1::EGFP::3xFLAG(92C12)+unc-119(+)]*), N2 Bristol, PS3351 (*hsf-1(sy441)*), TU3401 (*uIs69[pCFJ90 (myo-2p::mCherry+unc-119p::SID-1]*), NL3321 (*sid-1(pk3321)*), KP4 (*glr-1(n2461)*), CX13079 *(octr-1(ok3710))*, DA1814 *(ser-1(ok345))*, and CL2006 (*dvIs2 [pcL12(unc-54/human Abeta peptide minigene)+pRF4*] were obtained from the *Caenorhabditis* Genetics Center. The Q35 Huntington's Disease model was a gift from Dr. Richard I. Morimoto (AM167 *(rmIs156[unc-54p::Q35::YFP*].
C. elegans strains used for tissue-specific overexpression of HSP-90 (DAF-21) were as described in (van Oosten-Hawle et al., 2013), either overexpressing *HSP-90::GFP^{neuro}* in the neurons (AM778 *(rmIs314[F25B3.3p::DAF-21::GFP]; pCFJ90[myo-2p::RFP]*) or *HSP-90::RFP* in the neurons *HSP-90::RFP^{neuro}* (AM987 (*rmIs345[F25B3.3p::DAF-21::RFP*] or the intestine *HSP-90::RFP^{int}* (AM986 *rmIs346[vha-6p::DAF-21::RFP]),* or the bodywall muscle *HSP-90::RFP^{bwm} (AM988 (rmIs347(unc-54p::DAF-21::RFP].* The *hsp-90* reporter strain (AM799 (*rmIs317[Pdaf-21::GFP, pCeh361*] was used as a read-out for transcriptional activity of *hsp-90* in the different tissues. Strains overexpressing *HSP-90::RFP* in the neurons (*HSP-90::RFP^{neuro}*) and intestine *HSP-90::RFP^{int}* or the bodywall muscle *HSP-90::RFP^{muscle}* were crossed into the *hsp-90* reporter strain resulting in (AM1011 (*rmIs345[F25B3.3p::DAF-21::RFP]; rmIs317[Pdaf-21::GFP, pCeh361*]); and AM1010 (*rmIs346[vha-6p::DAF-21::RFP]; rmIs317[Pdaf-21::GFP, pCeh361*]), or AM1012 (*rmIs345[unc-54p::DAF-21::RFP]; rmIs317[Pdaf-21::GFP, pCeh361*]) respectively) as described previously (van Oosten-Hawle et al., 2013).

The A$\beta_{(3-42)}$ expressing strain (CL2006) was crossed into the genetic background of strains overexpressing *HSP-90::RFP* in the neurons, intestine or bodywall muscle, resulting in strains PVH85 (*rmIs345[F25B3.3p::DAF-21::RFP]; dvIs2),* PVH127 (*rmIs346[vha-6p::DAF-21::RFP]; dvIs2)*, and PVH50 *(*AM988 *(rmIs347(unc-54p::DAF-21::RFP]; dvIs2),* respectively.

The *pqm-1 (ok435)* knockout mutant (RB711) was crossed into strains AM799, AM1011 and AM1010 and AM1012 to result in strains PVH72 (*rmIs317[daf-21p::GFP, pCeh361], pqm-1(ok435)),* PVH68 (*rmIs346[vha-6p::DAF-21::RFP]; rmIs317[Pdaf-21::GFP, pCeh361*]; *pqm-1(ok435))* and PVH55 (*rmIs345[F25B3.3p::DAF-21::RFP]; rmIs317[Pdaf-21::GFP, pCeh361*], *pqm-1(ok435))* and PVH128 *(rmIs346[unc-54p::DAF-21::RFP]; rmIs317[Pdaf-21::GFP, pCeh361*]; *pqm-1(ok435))* respectively. The *pqm-1(ok435)* mutant was also crossed into AM167 to result in PVH45 *(rmIs156[unc-54p::Q35::YFP; pqm-1(ok435)*], and crossed into the *hsf-1(sy441)* mutant to result in PVH46 (*hsf-1(sy441); pqm-1(ok435)*).

To test which neuronal signalling pathway might be involved via neuron-induced TCS, strain AM1011 was crossed into the genetic background of the *glr-1(n2461)* mutant KP4, or *ser-1(ok345)* mutant DA1814 or *octr-1(ok371)* mutant CX13079 resulting in strains PVH135 ((*rmIs345[F25B3.3p::DAF-21::RFP]; rmIs317[Pdaf-21::GFP, pCeh361];glr-1(n2461);* PVH136 ((*rmIs345[F25B3.3p::DAF-21::RFP]; rmIs317[Pdaf-21::GFP, pCeh361];ser-1(ok345)) or* PVH138 ((*rmIs345[F25B3.3p::DAF-21::RFP]; rmIs317[Pdaf-21::GFP, pCeh361];octr-1(ok371)).*

Strains overexpressing *HSP-90::RFP* in the neurons (AM987) or the intestine (AM986) or the muscle (AM988) were crossed with OP201 to result in strains PVH58 (*rmIs345[F25B3.3p::DAF-21::RFP*]; *wgIs201)* and PVH64 (*rmIs346[vha-6p::DAF-21::RFP]; wgIs201)* and PVH145 *rmIs346[unc-54p::DAF-21::RFP]; wgIs201).*

RNAi insensitive strains and strains sensitive to RNAi in specific tissues created in this study were: PVH26 (*rmIs345[F25B3.3p::DAF-21::RFP];rmIs317[Pdaf-21::GFP,pCeh361*];*sid-1(pk3321))*; PVH14 (*rmIs346[vha-6p::DAF-21::RFP]; rmIs317[Pdaf-21::GFP, pCeh361*]; *sid-1(pk3321))* and PVH16 (*rmIs345[F25B3.3p::DAF-21::RFP];rmIs317[Pdaf-21::GFP,pCeh361*];*sid-1(pk3321);uIs69*); PVH17 (*rmIs345[F25B3.3p::DAF-21::RFP];rmIs317[Pdaf-21::GFP,pCeh361*];*sid-1(pk3321); pccIs04[pCFJ90 (myo-2p::mCherry+vha-6p::SID-1]);* PVH19 (*rmIs346[vha-6p::DAF-21::RFP]; rmIs317[Pdaf-21::GFP, pCeh361*]; *sid-1(pk3321) uIs69*); PVH20 (*rmIs346[vha-6p::DAF-21::RFP]; rmIs317[Pdaf-21::GFP, pCeh361*]; *sid-1(pk3321), pccIs04);* PVH18 *(rmIs345[F25B3.3p::DAF-21::RFP];rmIs317[Pdaf-21::GFP,pCeh361*];*pccIs005[myo-3p::SID-1::unc-54 3'UTR +myo-2p::RFP]; sid-1 (pk3321));* PVH21 (*rmIs346[vha-6p::DAF-21::RFP]; rmIs317[Pdaf-21::GFP, pCeh361*]; *pccIs005[myo-3p::SID-1::unc-54 3'UTR +myo-2p::RFP]; sid-1 (pk3321));* PVH15 *rmIs347[unc-54p::DAF-21::RFP]; rmIs317[daf-21pr::GFP; pCeh361]; sid-1(pk3321);* PVH129

rmIs347[unc-54p::DAF-21::RFP]; rmIs317[daf-21pr::GFP; pCeh361]; sid-1(pk3321) uIs69 [pCFJ90 (myo-2p::mCherry) + unc-119p::sid-1]; PVH146 *rmIs347[unc-54p::DAF-21::RFP]; rmIs317[daf-21pr::GFP; pCeh361]; sid-1(pk3321)* pccIs004[vha-6p::SID-1::unc-54 3'UTR + myo-2p::RFP] *and* PVH147 *rmIs347[unc-54p::DAF-21::RFP]; rmIs317[daf-21pr::GFP; pCeh361]; sid-1(pk3321)pccIs005[myo-3p::SID-1::unc-54 3'UTR +myo-2p::RFP].*
To deplete neurotransmitter and neuropeptide signalling simultaneously, strains PVH16, PVH19 and PVH129 allowing for neuron-specific RNAi, were crossed into the genetic background of *unc-31(e928)* mutants (strain CB928), resulting in strains PVH148, PVH149 and PVH150 respectively.

Generation of *hsp-90* (*daf-21*) transcriptional GFP reporters containing or deleted for the putative PQM-1 binding motif

The *hsp-90* promoter region (1,128 bp upstream of the start codon) was amplified and cloned, using *MScI* and *BamHI* restriction sites, into a pPD95.75 vector which had been modified to contain *Cbr-unc-119* (Marvin, 2015). A modified version of the promoter (purchased from Eurofins, UK) containing a 10 bp deletion which corresponds to the predicted PQM-1 binding site (119-129 upstream of the start codon), was also cloned into the same vector. DNA sequencing was performed (GATC Biotech, UK) prior to *C. elegans* transformation. Clones were linearised with *EagI* and microprojectile bombardments were performed using the Bio-Rad PDS-1000/He with Hepta adapter as previously described (Dupuy et al., 2004). Five stable strains were isolated for each construct and three strains for each construct were crossed into AM987 *(HSP-90::RFP^{neuro})*. GFP expression was assessed in five hermaphrodites on the first day of adulthood for each strain using an inverted Zeiss LSM 510 META Axiovert 200M and Image Pro Analyser 7.0 (Media Cybernetics).

RNA-Seq Analysis

The experiment was performed with three biological replicates of wild type and *HSP-90::GFP^{neuro}*, grown at 20°C. Eggs were synchronized to L1 larvae overnight in M9 and 1000 larvae were grown to L4 on NGM seeded with OP50. Animals were collected and washed three times with M9 media to remove bacteria. Worms were then snap frozen in liquid nitrogen. RNA was extracted using TriZol and then purified using the RNeasy mini kit (QIAGEN). RNA quality was measured using an Agilent Technologies 2100 Bioanalyzer. All samples had an RNA integrity number of > 9.5. cDNA libraries were prepared from 5 µgs of total RNA using the TruSeq RNA Sample Preparation v2 kit (Illumina). 50 cycle single-end sequencing was performed on an Illumina HiSeq 2000 by the IGSB Sequencing Core (Institute for Genomics and Systems Biology, University of Chicago, U.S.A). RNA-Seq analysis was performed by ContigExpress (contigexpress.com). Read quality on the raw sequencing data was performed with FastQC. Reads were aligned to the *C. elegans* genome (version WS220). Reference sequences were indexed using Bowtie2 (version 2.0.0-beta7) and the raw reads were mapped using Tophat (version 2.0.4). Cufflinks (version 2.0.2) was used for read quantification and differential expression analysis between wild type and the *C. elegans* strain overexpressing *HSP-90::GFP* in the neurons (*HSP-90::GFP^{neuro}*). Differentially expressed genes were identified with the p-adj cut-off 0.05.

Motif Discovery

Ab initio and known motif discovery was performed using HOMER (Hypergeometric Optimization of Motif EnRichment) (Benner et al., 2017). Target sequences were defined from 1000 bases upstream of the transcription start site for all up-regulated genes in *HSP-90::GFP^{neuro}*. Statistically enriched motifs were identified, with the PQM-1 motif significantly enriched in overall up-regulated genes in *HSP-90::GFP^{neuro}* ($P = 1e\text{-}9$).

Gene Ontology, signal peptide analysis and target compartment prediction analysis

Entries of the 34 significantly upregulated genes (Table S2) were manually curated for Gene Ontology classification on wormbase (www.wormbase.org). Putatively secreted peptides were analysed for the presence of signal peptides using SignalIP (www.cbs.dtu.dk/services/SignalIP) as described in Petersen et al. (Petersen et al., 2011) and for target compartments using WoLF PSORT (https://wolfpsort.hgc.jp) as described in Horton et al. (Horton et al., 2007).

Quantitative RT-PCR primers used

cdc-42 forward
5'-TGTCGGTAAAACTTGTCTCCTG-3'

cdc-42 reverse
5'-ATCCTAATGTGTATGGCTCGC-3'

hsp-90 forward
5'- GACCAGAAACCCAGACGATATC -3'

hsp-90 reverse
5'- GAAGAGCACGGAATTCAAGTTG -3'

GFP forward
5'-CCACATGGTCCTTCTTGAGTTT-3'

GFP reverse
5'- ATAGTTCATCCATGCCATGTGTA-3'

hsp-70 (C12C8.1) forward
5'- CTACATGCAAAGCGATTGGA -3'

hsp-70 (C12C8.1) reverse
5'- GGCGTAGTCTTGTTCCCTTC – 3'

hsp-1 forward
5'-AAGCCGCTCAAAAAATGTCG-3'

hsp-1 reverse
5'-GGCCAATCCTTCCAAATCCTTCTG-3'

hsp-16.2 forward
5'-TCCATCTGAGTCTTCTGAGATTGTTA-3'

hsp-16.2 reverse
5'-TGGTTTAAACTGTGAGACGTTGA-3'

pqm-1 forward
5'-TCCAGAGAGTATCGACCAAGG-3'

pqm-1 reverse
5'-TCATTGATATTTGATAAGCCATCTTT-3'

clec-41 forward
5'-GATCAAATGTTCTTTGTCTGGA-3'

clec-41 reverse
5'-TCCAACTTCAACAAGAGGGTATC-3'

ckb-2 forward
5'-CCTGGAATCAAGATGAATGGA-3'

ckb-2 reverse
5'-ATGGTGAACGGTTTTTGAGC-3'

asp-12 forward
5'-CGGAGATGGATGACATTTGAG-3'

asp-12 reverse
5'-ACCACAGTTGCCGAGCAC-3'

Microscopy and Fluorescence Image Quantification

C. elegans was imaged using a Zeiss LSM880 confocal microscope through a 10x 1.0 or a 20x 1.0 numerical aperture objective with a 488 nm line for excitation of GFP and 587 nm line for excitation of mCherry (RFP). For imaging, age-synchronised L4 animals or Day 1 adult animals (L4 + 24 hours) were anesthesised using 5 mM Levamisole solution in M9 buffer and mounted on 2% agar pads. Quantification of *hsp-90p::GFP* or *HSP-90::RFP* fluorescence intensity was performed using ImageJ software of three biological replicate images (n > 15). Fluorescence intensity was calculated as pixels per unit area, and background fluorescence subtracted. Intensities were normalised to untreated controls.

Western Blot Analysis and Quantification of Protein levels

For Western Blot analysis, cell extracts were prepared of 10.000 age-synchronised animals grown on 10 cm NGM plates at a population density of 1000 worms per plate. Young adult animals were harvested into a worm pellet of 200 ul and flash frozen in liquid nitrogen. The frozen pellet was ground with an Eppendorf pestle and re-suspended in Worm Lysis Buffer (10 mM Tris pH 7.5; 150 mM NaCl; 0.5 mM EDTA; 0.5% NP-40; 1 mM PMSF), supplemented with EDTA-free protease inhibitor cocktail tablet (Complete Mini, EDTA-free, Roche). The cell extract was prepared by centrifugation at 10 000 x g for 5 minutes at 4°C and protein concentration was determined using the Bio Rad protein assay kit (Bradford assay). Cell extracts were mixed with 5x SDS sample buffer and boiled for 5 min. 25 ug total protein was loaded onto a 10 % SDS-PAGE and western blot analysis was performed as described previously (van Oosten-Hawle et al., 2013). To detect *C. elegans* endogenous HSP-90 or the GFP or RFP-tagged version, a poly clonal anti-*C.e. HSP-90* antibody raised in rabbit was used (van Oosten-Hawle et al., 2013). A monoclonal mouse anti-tubulin antibody (Sigma) was used to detect tubulin as a loading control. HRP-conjugated anti-mouse or anti-rabbit antibodies were used as secondary antibodies and ECL reagent (Thermo Fisher Scientific) was used for detection. A monoclonal anti-FLAG antibody (Sigma) was used to detect PQM-1::GFP::FLAG.
The gel analysis tool of ImageJ software was used to quantify HSP-90::GFP or HSP-90::RFP relative to endogenous HSP-90 levels and normalised to tubulin.

Western Blotting of Aβ species

The Aβ species of *C. elegans* expressing Aβ($_{3\text{-}42}$) in the bodywall muscle (CL2006) or in strains overexpressing HSP-90 in the muscle (PVH50), intestine (PVH127) or neurons (PVH85) was identified by immunoblotting using a 16% Tris-Tricine gel containing 6M urea, and the standard Western blotting protocol, except that the 0.2 um nitrocellulose membranes were boiled in PBS buffer for 5 min after the transfer. 200 nematodes per strain were collected at Day 3 of adulthood into ice-cold M9 buffer, washed in M9 and flash frozen in liquid nitrogen. Nematodes were lysed by grinding using the following lysis buffer: 62 mM Tris-HCl pH 6.8, 5% b-mercaptoethanol, 10% glycerol, 2% SDS, and 1x protease inhibitor cocktail tablet (Roche). 50 ug of total protein for each sample was loaded onto the 16% Tris-Tricine gel. Amyloid monomeric and oligomeric species were detected with 6E10 monoclonal antibody (1:500, Absolute Antibody). Tubulin (anti-tubulin antibody; Sigma) was used as a loading control.

***P. aeruginosa* (PA14) survival assays**

P. aeruginosa cultures were grown in LB medium at 37°C overnight. Bacterial lawns used for *C. elegans* killing assays were prepared by spreading 5 ul of an overnight culture of the bacterial strain on modified NGM agar plates (50 mM NaCl, 0,35% peptone) in 3 cm diameter plates. Plates were incubated 24h at 25°C before seeding with L4 *C. elegans* grown on OP50 plates. The killing assays were performed at 20°C and animals were scored and transferred each day to fresh plates. Animals were considered dead when they failed to respond to the touch-nose-response.

Heat Shock experiments

Synchronised populations of 30 *C. elegans* animals were grown at 20°C and heat-shocked for 1 hour in a water bath equilibrated at 35°C and allowed to recover for 1 hour at 20°C before they were collected for quantitative RT-PCR, as previously described (van Oosten-Hawle et al., 2013). Each heat shock experiment and following qRT-PCR was performed in triplicate.

Thermotolerance assays

Synchronised populations of 100 L4-staged animals were placed on a NGM-Agar plate seeded with *OP50-1 E. coli* and incubated in a water bath equilibrated at 35°C for indicated time points. 4 samples, each consisting of 25-30 L4 animals were used for one time point. After collection of the plates at the indicated time points, animals were allowed to recover for 16 hours at 20°C before scoring for touch-induced movement and pharyngeal pumping. Each experiment was repeated five times.

Paralysis assays

An age-synchronised population of 100 animals per strain was scored by monitoring their movement via the touch-nose response, using a platinum wire. Animals were transferred to fresh OP50-1 plates or indicated RNAi plates every day.

Quantification of Q35::YFP aggregates

Age-synchronised *C. elegans* (n > 30) expressing *unc-54p::Q35::YFP* (*mQ35*) were imaged using a Leica MZ10F fluorescent stereoscope with a YFP filter and the number of aggregates was counted each day of adulthood (L4 = Day 0). Aggregates defined as fluorescent foci were brighter and clearly distinguishable from background YFP fluorescence (Morley et al., 2002).

Subcellular localisation of PQM-1::GFP::FLAG

Each strain was age-synchronised via egg laying and > 20 animals were imaged at a confocal fluorescent microscope (LSM880) at 20x and scored blindly for clearly visible nuclear or cytosolic localisation of GFP. Statistical analysis was performed using a Kruskal-Wallis test.

SUPPLEMENTAL TABLES

Table S1. Differentially expressed genes in *C. elegans* overexpressing HSP-90::GFP in the neurons (*HSP-90::GFP^{neuro}*) relative to control animals (N2), at normal growth conditions (20°C). Related to Figure 2 and Figure S1.

Table S2. Genes upregulated > 1.5-fold in *C. elegans* overexpressing HSP-90::GFP in the neurons (*HSP-90::GFP^{neuro}*). Pseudogenes are indicated in red. Y=Yes; N=No. Related to Figure 2 and Figure S1.

Figure S1, related to Figure 1 and Figure 2

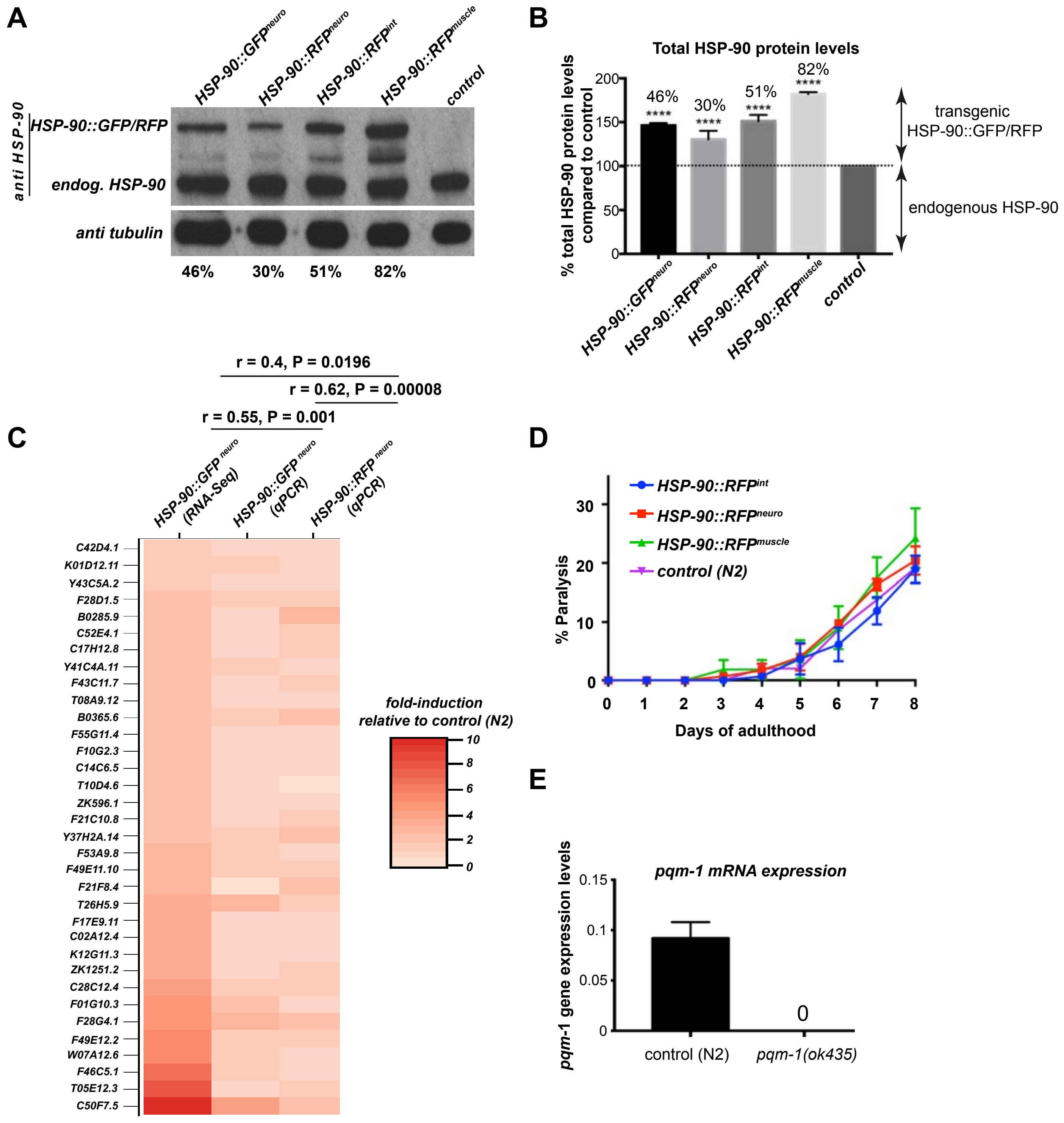

Figure S1. Analysis of *C. elegans* strains overexpressing HSP-90 protein in the neurons; Related to Figure 1 and 2. **(A)** Western blot analysis of Day 1 adult *HSP-90::GFP^{neuro}*, *HSP-90::RFP^{neuro}*, *HSP-90::RFP^{int}* or *HSP-90::RFP^{muscle}* using an anti-*C. elegans* HSP-90 antibody. **(B)** Quantification of total HSP-90 protein levels, corresponding to the sum of endogenous HSP-90 and transgenic HSP-90::GFP/RFP expressed in *HSP-90::GFP^{neuro}*, *HSP-90::RFP^{neuro}* and *HSP-90::RFP^{int}* compared to control (N2). HSP-90::GFP/RFP protein levels are normalised to the loading control (tubulin) and relative to endogenous HSP-90. **(C)** Heat-map of gene expression levels of genes induced > 1.5-fold in the RNA-seq analysis of *HSP-90::GFP^{neuro}* relative to control (N2). Analysis of expression levels of the 34 induced genes in *HSP-90::GFP^{neuro}* by qRT-PCR compared to *HSP-90::GFP^{neuro}* (RNA-Seq) revealed a medium-high correlation following Pearson correlation analysis ($r = 0.55, P = 0.001$; two-tailed t-test). qRT-PCR analysis of expression levels in *HSP-90::RFP^{neuro}* compared to the RNA-Seq analysis of *HSP-90::GFP^{neuro}* showed a medium correlation ($r = 0.4$; $P < 0.02$; two tailed t-test), and a high correlation between qRT-PCR analysis of induced genes between *HSP-90::RFP^{neuro}* and *HSP-90::GFP^{neuro}* ($r = 0.62$, $P = 0.00008$). **(D)** Paralysis assays of *HSP-90::RFP^{neuro}*, *HSP-90::RFP^{int}* or *HSP-90::RFP^{muscle}* compared to wild type (N2) animals. **(E)** *pqm-1* transcript levels in wild type control animals (N2) and the *pqm-1(ok485)* deletion mutant.

Figure S2, related to Figure 2

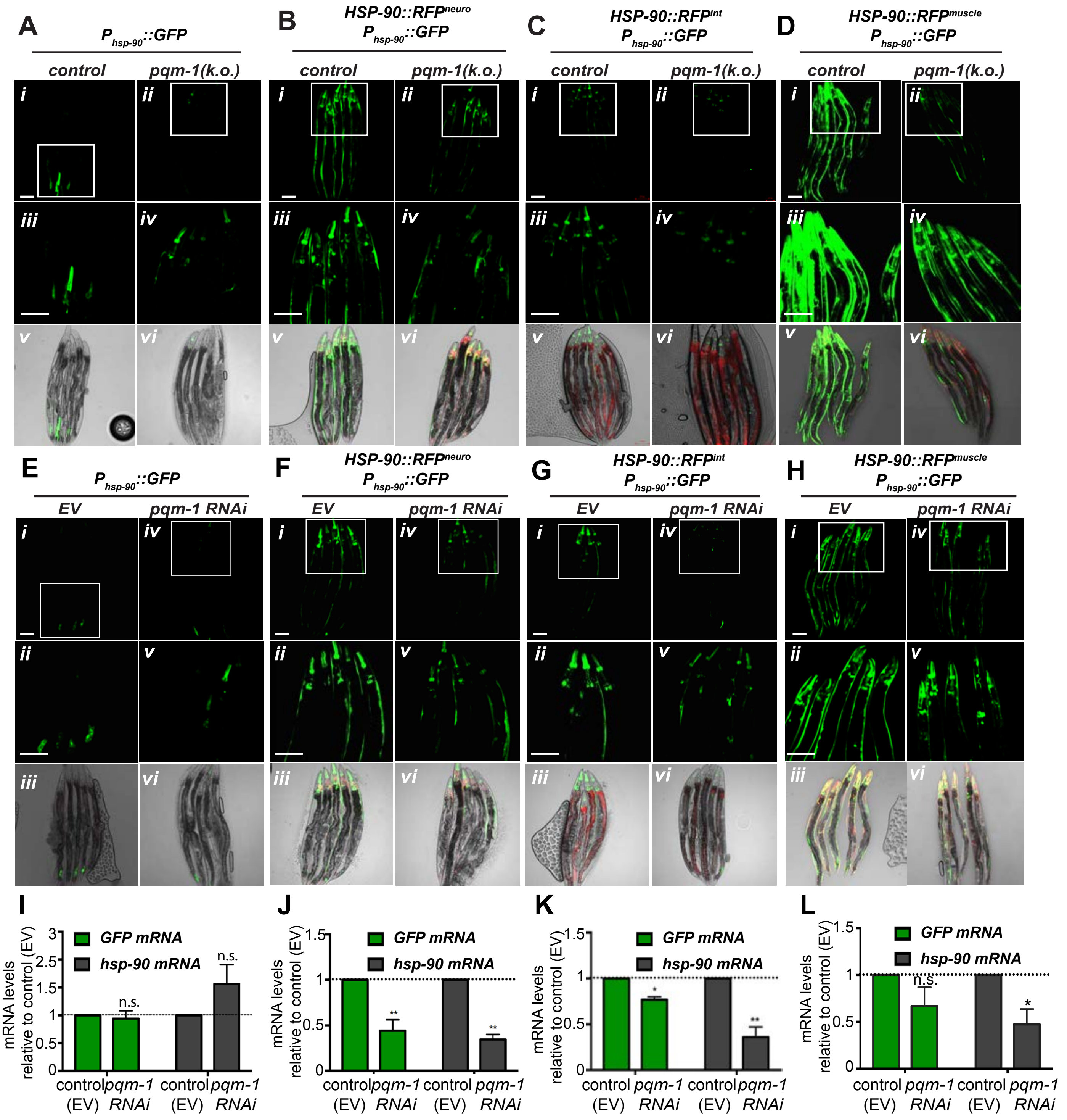

Figure S2. Depletion of *pqm-1* reduces TCS-induced *hsp-90* expression in distal tissues; Related to Figure 2. Depletion of *pqm-1* reduces TCS-induced *hsp-90* expression in distal tissues. P_{hsp-90}*::GFP* expression **(A i, iii, v)** in control animals or **(A ii, iv, vi)** crossed into a *pqm-1* knockout (*k.o.*) mutant (*pqm-1(ok435)*); **(B i, iii, v)** in *HSP-90::RFP*^{neuro}; or **(B ii, iv, vi)** *HSP-90::RFP*^{neuro}*;pqm-1(k.o.)* and **(C i, iii, v)** in *HSP-90::RFP*^{int} or **(C ii, iv, vi)** *HSP-90::RFP*^{int}*;pqm-1(k.o.)*, or in **(D i, iii, v)** *HSP-90::RFP*^{muscle} or **(D ii, iv, vi)** *HSP-90::RFP*^{muscle}*;pqm-1(k.o.)*. **(A,B,C,D iii and iv)** 20 x magnification of **(A iii)** tail region or **(A iv; B, C,D iii and iv)** head region. Scale bar, 50 um. **(E-H)** P_{hsp-90}*::GFP* expression in **(E, i)** wild type animals treated with empty vector (EV) control RNAi or **(E, iv)** with *pqm-1* RNAi. P_{hsp-90}*::GFP* expression **(F, i)** in *HSP-90::RFP*^{neuro} during EV control RNAi or **(F, iv)** *pqm-1* RNAi; **(G, i)** P_{hsp-90}*::GFP* expression in *HSP-90::RFP*^{int} during EV RNAi or **(G, iv)** *pqm-1* RNAi. **(H, i)** P_{hsp-90}*::GFP* expression in *HSP-90::RFP*^{muscle} during EV RNAi or **(G, iv)** *pqm-1* RNAi. **(E,F,G,H, ii and v)** 20x magnification of the anterior (head) region of **(E, ii)** wild type, **(F, ii)** *HSP-90::RFP*^{neuro} and **(G, ii)** *HSP-90::RFP*^{int} and **(H, ii)** *HSP-90::RFP*^{muscle} animals expressing the P_{hsp-90}*::GFP* reporter during EV control RNAi and **(E, v)** wild type, **(F, v)** *HSP-90::RFP*^{neuro} and **(G, v)** *HSP-90::RFP*^{int} and **(H, v)** *HSP-90::RFP*^{muscle} expressing the P_{hsp-90}*::GFP* reporter treated with *pqm-1* RNAi. **(E-H, iii)** Differential interference contrast (DIC) Normarski images. Scale bar, 50 um. **(I - L)** GFP and *hsp-90* transcript levels in *C. elegans* expressing the P_{hsp-90}*::GFP* reporter during *pqm-1* RNAi, relative to control (EV) RNAi in **(I)** wild type animals expressing the P_{hsp-90}*::GFP* reporter **(J)** in *HSP-90::RFP*^{neuro};P_{hsp-90}*::GFP* or **(K)** in *HSP-90::RFP*^{int};P_{hsp-90}*::GFP* and **(L)** in *HSP-90::RFP*^{muscle};P_{hsp-90}*::GFP*.

Figure S3; Related to Figure 3 and Figure 4

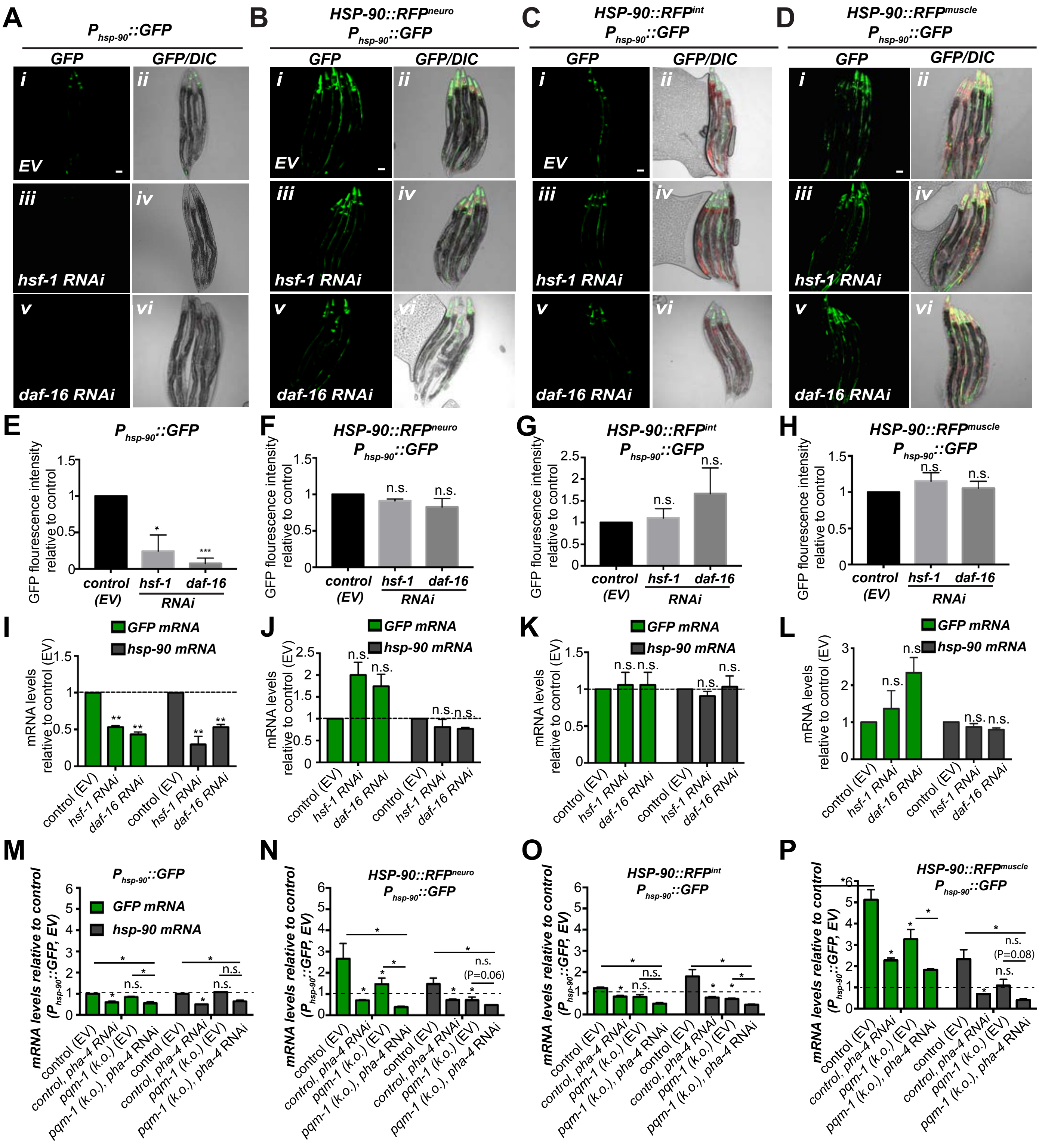

Figure S3. RNAi-mediated knockdown of stress transcription factors *hsf-1* or *daf-16* does not affect TCS-induced *hsp-90* expression; Related to Figure 3. P_{hsp-90}*::GFP* expression in **(A, i)** wild type animals treated with empty vector (EV) control RNAi or **(A, iii)** *hsf-1* RNAi or **(A, v)** *daf-16* RNAi. **(B, i)** P_{hsp-90}*::GFP* expression in *HSP-90::RFP*^{neuro} during control RNAi or **(B, iii)** during *hsf-1* RNAi or **(B, v)** *daf-16* RNAi. **(C, i)** P_{hsp-90}*::GFP* expression in *HSP-90::RFP*^{int} during control RNAi or **(C, iii)** *hsf-1* RNAi or **(C, v)** *daf-16* RNAi. **(D, i)** P_{hsp-90}*::GFP* expression in *HSP-90::RFP*^{muscle} during control RNAi or **(D, iii)** *hsf-1* RNAi or **(D, v)** *daf-16* RNAi. **(A, B, C, and D ii, iv, vi)** Differential interference contrast (DIC) Nomarski images. Scale bar, 50 um. **(E-H)** Quantification of P_{hsp-90}*::GFP* fluorescence intensity in **(E)** control (P_{hsp-90}*::GFP*), **(F)** *HSP-90::RFP*^{neuro};P_{hsp-90}*::GFP* and **(G)** *HSP-90::RFP*^{int};P_{hsp-90}*::GFP* and **(H)** *HSP-90::RFP*^{muscle};P_{hsp-90}*::GFP* animals during *hsf-1* or *daf-16* RNAi compared to control RNAi. Error bars represent +/- SEM of three biological replicates. (n.s.) non-significant. $^*P < 0.05$; $^{***}P < 0.001$. **(I - L)** GFP and *hsp-90* transcript levels in *C. elegans* expressing the P_{hsp-90}*::GFP* reporter during *hsf-1* or *daf-16* RNAi, relative to control (EV) RNAi in **(I)** wild type animals expressing the P_{hsp-90}*::GFP* reporter **(J)** in *HSP-90::RFP*^{neuro};P_{hsp-90}*::GFP*; **(K)** in *HSP-90::RFP*^{int};P_{hsp-90}*::GFP* or **(L)** *HSP-90::RFP*^{muscle};P_{hsp-90}*::GFP*. **(M – P)** *pha-4* RNAi further reduces GFP and *hsp-90* transcript levels in **(N)** *HSP-90::RFP*^{neuro};P_{hsp-90}*::GFP;pqm-1(k.o.)*, **(O)** *HSP-90::RFP*^{int};P_{hsp-90}*::GFP;pqm-1(k.o.)* or **(P)** *HSP-90::RFP*^{muscle};P_{hsp-90}*::GFP;pqm-1(k.o.)*compared to control (EV) RNAi. **(M)** *pha-4* RNAi reduces GFP and *hsp-90* transcripts in the P_{hsp-90}*::GFP* control strain but not in P_{hsp-90}*::GFP;pqm-1(k.o.)* animals. **(I-P)** Bar graphs represent the combined results of three independent experiments. Error bars represent +/- SEM. $^*P < 0.05$; $^{**}P < 0.01$; (n.s.) non-significant.

Figure S4; Related to Figure 3 and 4

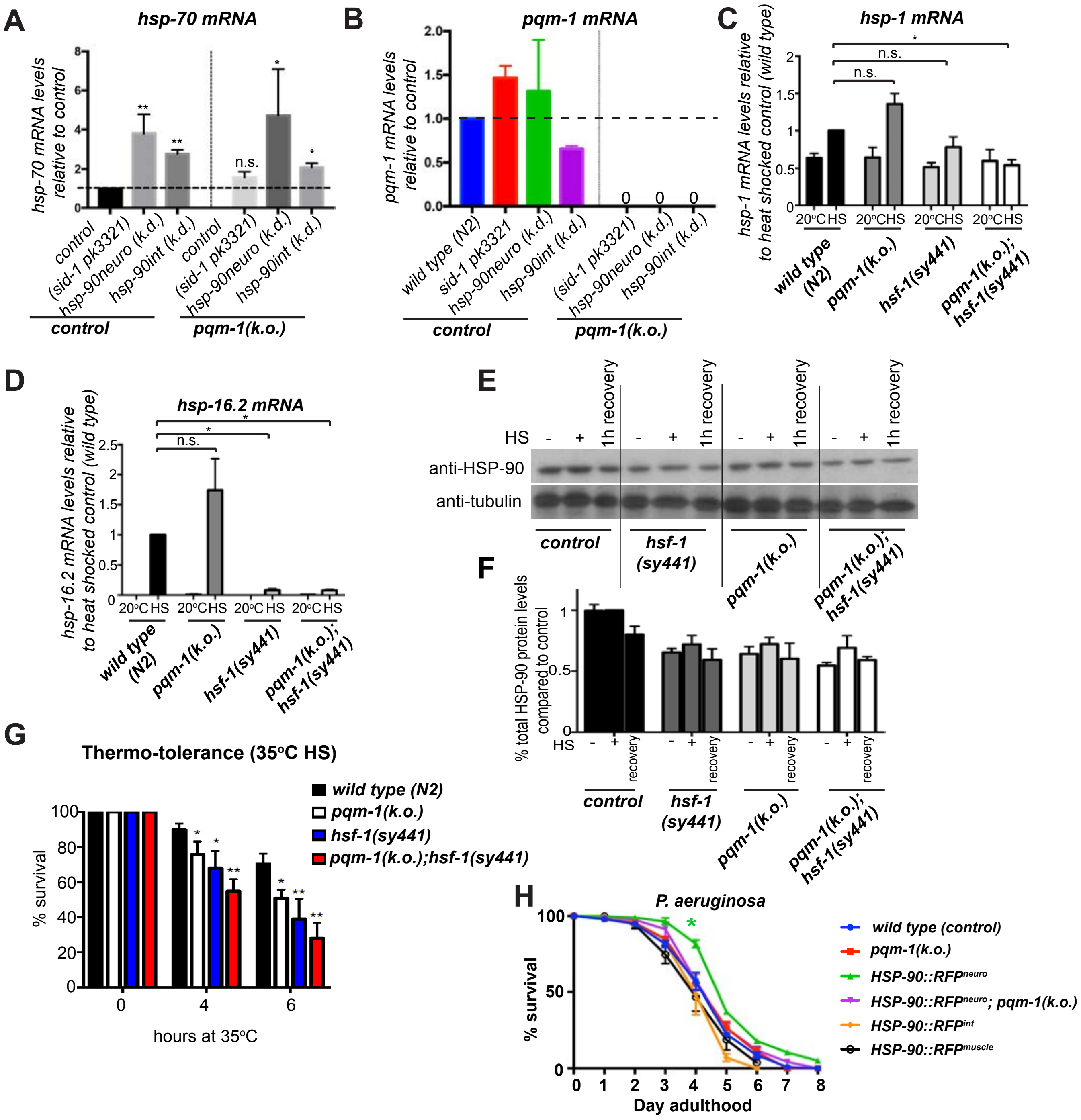

Figure S4. PQM-1 is required for heat-inducible expression of *hsp-90* but not heat- or TCS-induced expression of *hsp-70;* Related to Figure 3 and 4.(A and B) TCS-induced *hsp-70 (C12C8.1)* levels are not affected in tissue-specific *hsp-90* hairpin RNAi strains depleted for *pqm-1 (hsp-90neuro (k.d.)* and *hsp-90int (k.d.)). **(A)** hsp-70* mRNA levels are induced in *(hsp-90neuro (k.d.)* and *hsp-90int (k.d.)* compared to the control strain *(sid-1(pk3321))* and unaffected when crossed into the *pqm-1(k.o.)* mutant background. **(B)** *pqm-1* mRNA expression levels in *sid-1(pk3321)* mutants, *hsp-90neuro (k.d.)* and *hsp-90int (k.d.)* strains compared to N2 animals. No *pqm-1* mRNA transcripts were measured in *sid-1(pk3321);pqm-1(k.o)* mutants, *hsp-90neuro(k.d.);pqm-1(k.o.)* or *hsp-90int(k.d.);pqm-1(k.o.)* strains harbouring the *pqm-1(ok435)* deletion mutation.
Total mRNA accumulation of **(C)** *hsp-1* (constitutive hsp70) and **(D)** heat-inducible *hsp-16.2* before (20°C) and after heat shock (HS; 1h at 35°C), in Day 1 adult wild type, *pqm-1(k.o.)* mutant, *hsf-1(sy441)* mutant, and *pqm-1(k.o.);hsf-1(sy441)* double mutant relative to heat shocked wild type animals. **(A - D)** Bar graphs represent combined means of three biological replicate experiments. Error bars represent +/- SEM. *P < 0.05; **P < 0.01; n.s. = not significant. **(E)** Western blot analysis of HSP-90 protein expression at 20°C (-), immediately after a 1h 35°C HS (+), and after a 1h recovery period at 20°C in young adult control animals, *hsf-1(sy441)* mutant, *pqm-1(k.o.)* mutant and *pqm-1(k.o.);hsf-1(sy441)* double mutants. **(F)** Quantification of HSP-90 levels. HSP-90 levels are normalised to the loading control (tubulin) and relative to HSP-90 levels in control animals at 20°C.
Bar graphs represent combined mean values of three independent experiments. Error bars represent +/- SEM.
(G) Thermo-sensitivity of L4 animals (n=100) with indicated genotypes after exposure to 35°C heat stress for 4 hours or 6 hours. Survival was measured after a recovery period of 16 hours at 20°C. *P < 0.05; **P < 0.01. Bar graphs represent combined mean values of four independent experiments. Error bars represent +/- SEM. **(H)** *HSP-90::RFP^{neuro}* require *pqm-1* for resistance to pathogenic challenge by *Pseudomonas aeruginosa*. *HSP-90::RFP^{int}* or *HSP-90::RFP^{muscle}* as well as the *pqm-1(k.o.)* mutant show similar survival curves as control animals (N2, wild type). Wilcoxon matched pairs-signed rank test; *P < 0.05.

Figure S5; Related to Figure 5

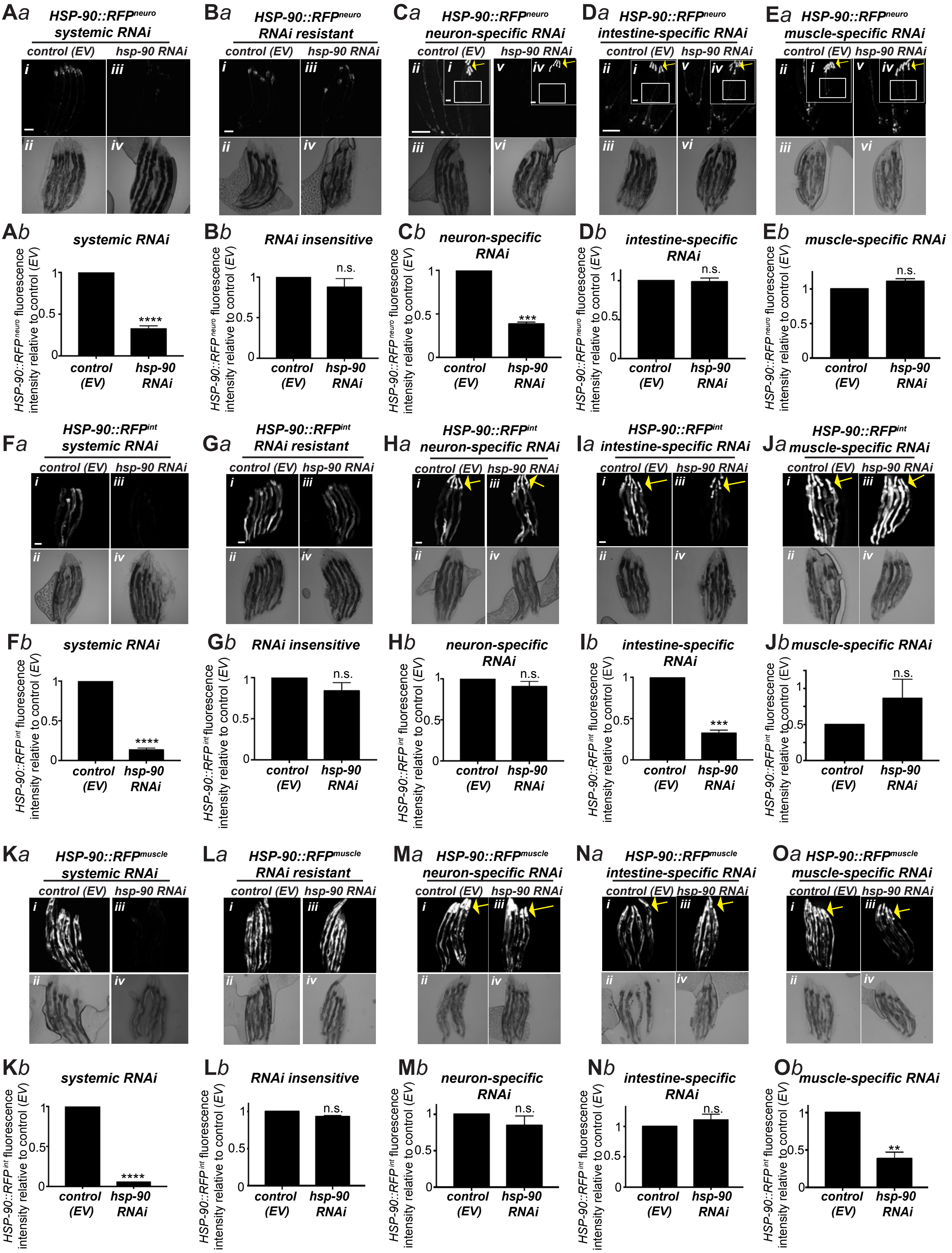

Figure S5. Analysis of systemic and tissue-specific *hsp-90* RNAi in *HSP-90::RFP^{neuro}*, *HSP-90::RFP^{int}* and *HSP-90::RFP^{muscle}*; Related to Figure 5.

(Aa) *HSP-90::RFP^{neuro}* animals treated with **(Aa, i and ii)** control (EV) RNAi or **(Aa, iii and iv)** *hsp-90* RNAi.

(Ba) RNAi-resistant *HSP-90::RFP^{neuro};P$_{hsp\text{-}90}$::GFP; sid-1(pk3321)* animals treated with **(Ba, i and ii)** control (EV) RNAi or **(Ba, iii and iv)** *hsp-90* RNAi. **(Ca)** HSP-90::RFP expression in the neurons during neuron-specific **(Ca, i, ii and iii)** control (EV) RNAi or **(Ca, iv, v, vi)** *hsp-90* RNAi in *HSP-90::;RFP^{neuro};P$_{hsp\text{-}90}$::GFP;sid-1(pk3321);unc-119p::SID-1* animals. **(Ca, ii and v)** 20x magnification of the posterior region. The posterior region was chosen to avoid interference with the pharyngeal *P$_{myo\text{-}2}$::mCherry* co-injection marker. **(Da)** H*SP-90::RFP^{neuro};P$_{hsp\text{-}90}$::GFP;sid-1(pk3321);vha-6p::SID-1* animals during intestine-specific **(Da, i, ii and iii)** control (EV) RNAi or **(Da, iv, v, vi)** *hsp-90* RNAi. **(Da, ii and v)** 20x magnification of the posterior region. **(Ea)** HSP-90::RFP expression during muscle-specific **(Ea, i, ii and iii)** control (EV) RNAi or **(Ea, iv, v, vi)** *hsp-90* RNAi in *HSP-90::RFP^{neuro};P$_{hsp\text{-}90}$::GFP;sid-1(pk3321);myo-3p::SID-1* animals. **(Ea, ii and v)** 20x magnification of the posterior region. **(Ca, Da, Ea)** Yellow arrow indicates a *P$_{myo\text{-}2}$::mCherry* co-injection marker. **(Fa)** *HSP-90::RFP^{int}* animals treated with **(Fa, i and ii)** systemic control (EV) RNAi or **(Fa, iii and iv)** *hsp-90* RNAi. **(Ga)** RNAi-resistant *HSP-90::RFP^{int}, P$_{hsp\text{-}90}$::GFP; sid-1(pk3321)* animals treated with **(Ga, i and ii)** control (EV) or **(Ga, iii and iv)** *hsp-90* RNAi. **(Ha)** HSP-90::RFP expression in *HSP-90::;RFP^{int};P$_{hsp\text{-}90}$::GFP, sid-1(pk3321);unc-119p::SID-1* animals, allowing neuron-specific **(Ha, i and ii)** control RNAi or **(Ha, iii and iv)** *hsp-90* RNAi.

(Ia) *HSP-90::RFP^{int};P$_{hsp\text{-}90}$::GFP, sid-1(pk3321); vha-6p::SID-1* animals allowing intestine-specific **(Ia, i and ii)** control (EV) RNAi or **(Ia, iii and iv)** *hsp-90* RNAi. **(Ja)** *HSP-90::RFP^{int};P$_{hsp\text{-}90}$::GFP, sid-1(pk3321); myo-3p::SID-1* animals allowing muscle-specific **(Ja, i and ii)** control (EV) RNAi or **(Ja, iii and iv)** *hsp-90* RNAi.

(Ka) *HSP-90::RFP^{muscle}* animals treated with **(Ka, i and ii)** systemic control (EV) RNAi or **(Ka, iii and iv)** *hsp-90* RNAi.

(La) RNAi-resistant *HSP-90::RFP^{muscle,} P$_{hsp\text{-}90}$::GFP; sid-1(pk3321)* animals treated with **(La, i and ii)** control (EV) or **(La, iii and iv)** *hsp-90* RNAi. **(Ma)** *HSP-90::RFP^{muscle};P$_{hsp\text{-}90}$::GFP, sid-1(pk3321);unc-119p::SID-1* animals, allowing neuron-specific **(Ma, i and ii)** control RNAi or **(Ma, iii and iv)** *hsp-90* RNAi.

(Na) *HSP-90::RFP^{muscle};P$_{hsp\text{-}90}$::GFP, sid-1(pk3321); vha-6p::SID-1* animals allowing intestine-specific **(Na, i and ii)** control (EV) RNAi or **(Na, iii and iv)** *hsp-90* RNAi.

(Oa) *HSP-90::RFP^{muscle};P$_{hsp\text{-}90}$::GFP, sid-1(pk3321); myo-3p::SID-1* animals allowing muscle-specific **(Oa, i and ii)** control (EV) RNAi or **(Oa, iii and iv)** *hsp-90* RNAi.

(Ab – Ob): Quantification of RFP fluorescence intensity in strains of the genotype indicated in **Aa – Oa**, respectively.

(Ca, Da, Ea, Ha, Ia, Ja, Ma, Na, Oa) Yellow arrow indicates a *P$_{myo\text{-}2}$::mCherry* co-injection marker.

(A-O) ***P < 0.001; ****P < 0.0001; n.s. = not significant. Scale bar, 50 um.

Bar graphs represent combined mean values of three independent experiments. Error bars represent +/- SEM.

Figure S6; Related to Figure 5

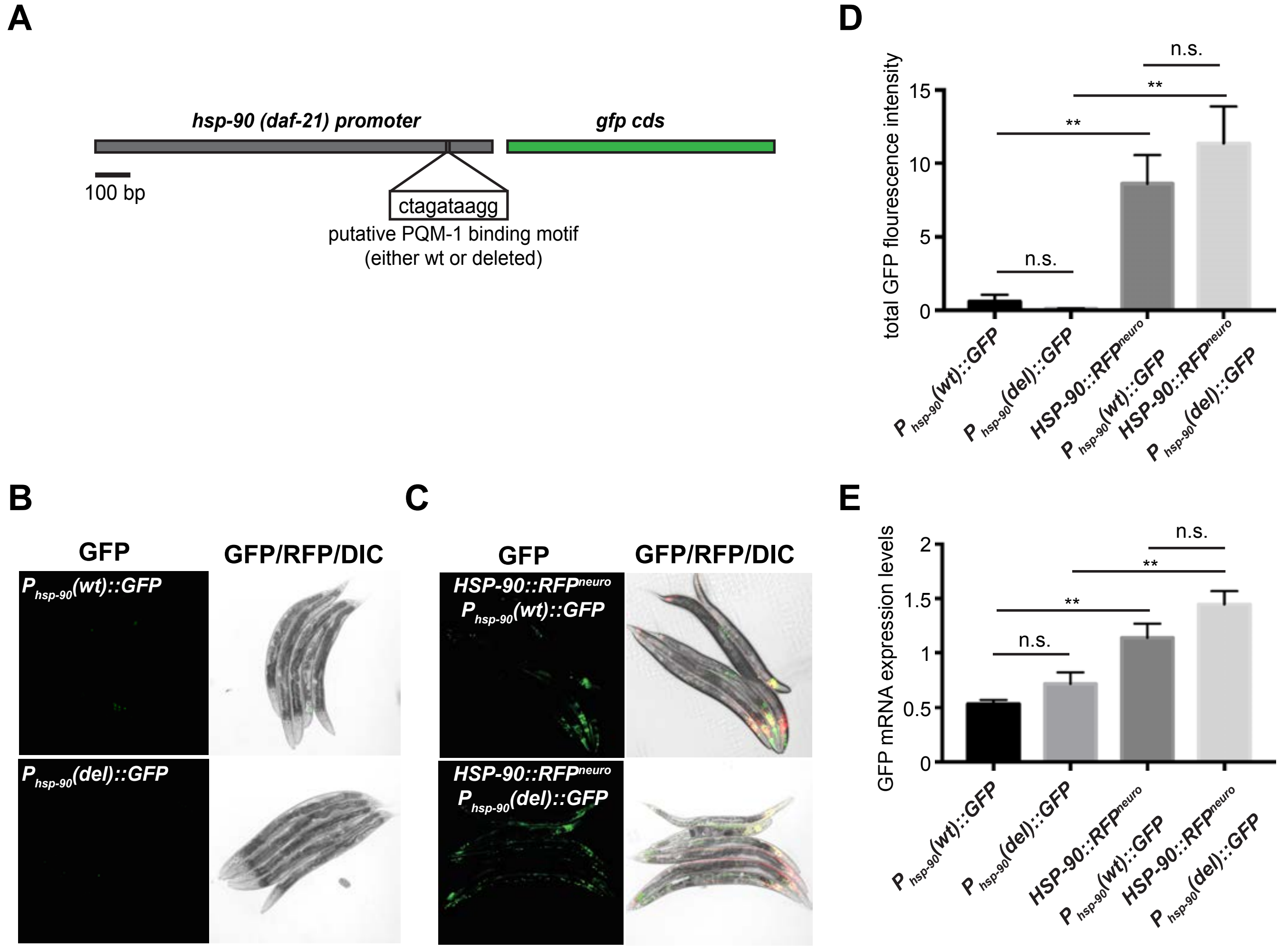

Figure S6. Deletion of the putative PQM-1 binding motif in the *hsp-90* promoter does not abolish TCS-induced and *pqm-1*-dependent $P_{hsp\text{-}90}$*::GFP* expression; Related to Figure 5 and Figure 6.
(A) Promoter reporter construct for $P_{hsp\text{-}90}$*::GFP* expression. The predicted PQM-1 binding motif is indicated.
(B) Day 1 adults expressing the low-copy $P_{hsp\text{-}90}$*::GFP* reporter construct, either containing the predicted PQM-1 motif ($P_{hsp\text{-}90}$*(wt)::GFP*) or a deleted motif ($P_{hsp\text{-}90}$*(del)::GFP*). **(C)** Day 1 adults expressing the $P_{hsp\text{-}90}$*(wt)::GFP* or $P_{hsp\text{-}90}$*(del)::GFP* reporter in the *HSP-90::RFP*^{neuro} strain background. **(D)** Quantification of total GFP fluorescence in $P_{hsp\text{-}90}$*(wt)::GFP* compared to $P_{hsp\text{-}90}$*(del)::GFP* and *HSP-90::RFP*^{neuro};$P_{hsp\text{-}90}$*(wt)::GFP* compared to *HSP-90::RFP*^{neuro};$P_{hsp\text{-}90}$*(del)::GFP*. n.s. = not significant. **(E)** GFP transcript levels in animals expressing the low-copy $P_{hsp\text{-}90}$*(wt)::GFP* reporter compared to the $P_{hsp\text{-}90}$*(del)::GFP* reporter construct and *HSP-90::RFP*^{neuro};$P_{hsp\text{-}90}$*(wt)::GFP* compared to *HSP-90::RFP*^{neuro};$P_{hsp\text{-}90}$*(del)::GFP*.
Bar graphs represent combined mean values of three independent experiments. Error bars represent +/- SEM. **$P < 0.01$

Figure S7; Related to Figure 6 and Figure 7

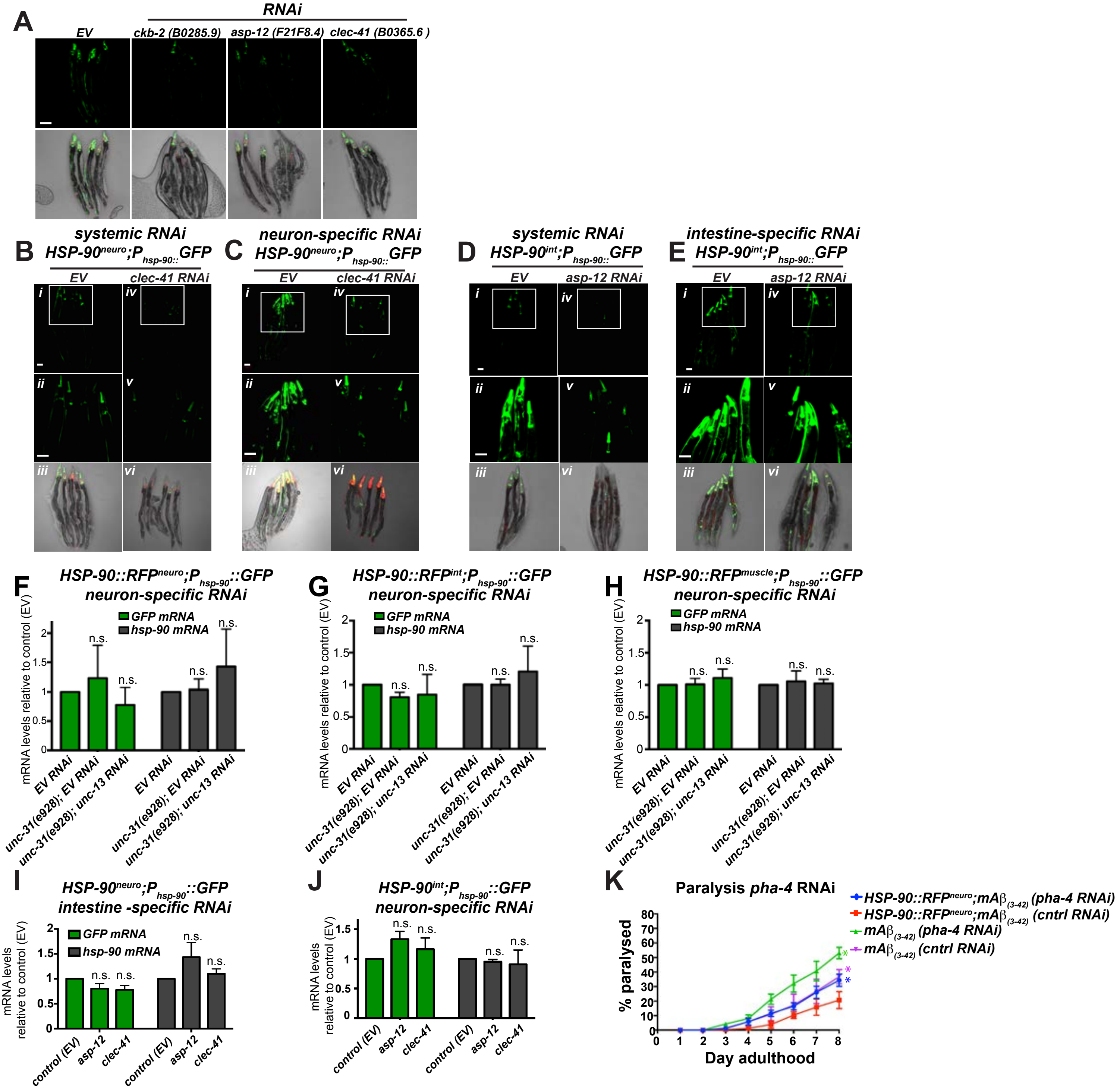

Figure S7. RNAi-mediated knockdown of genes involved in the innate immune response reduce TCS-induced *hsp-90* expression; Related to Figure 6. $P_{hsp\text{-}90}$*::GFP* expression in *HSP-90::RFP*^{neuro} fed with **(A)** control (EV) RNAi or *B0285.9, F21F8.4* or *B0365.6* RNAi. **(B)** *HSP-90::RFP*^{neuro};$P_{hsp\text{-}90}$*::GFP* animals treated with **(B, i and iii)** EV control RNAi or **(B, iv and vi)** *clec-41* RNAi. **(B, ii and v)** 20x magnification of the anterior (head) region and **(C)** *HSP-90::RFP*^{neuro};$P_{hsp\text{-}90}$*::GFP;sid-1(pk3321);unc-119p::SID-1* animals allowing for neuron-specific **(C, i and iii)** EV control RNAi or **(C, iv and vi)** *clec-41* RNAi. **(C, ii and v)** 20x magnification of the anterior (head) region. **(D)** *HSP-90::RFP*^{int};$P_{hsp\text{-}90}$*::GFP* animals treated with **(D, i and iii)** EV control RNAi or **(D, iv and vi)** *asp-12* RNAi. **(D, ii and v)** 20x magnification of the anterior (head) region. **(E)** *HSP-90::RFP*^{int};$P_{hsp\text{-}90}$*::GFP, sid-1(pk3321);vha-6p::SID-1* animals allowing for intestine-specific **(E, i and iii)** EV control RNAi or **(E, iv and vi)** *asp-12* RNAi. **(E, ii and v)** 20x magnification of the anterior (head) region. **(F - H)** Relative GFP and *hsp-90* mRNA levels in **(F)** *HSP-90::RFP*^{neuro};$P_{hsp\text{-}90}$*::GFP, sid-1(pk3321);unc-119p::SID-1,* **(G)** *HSP-90::RFP*^{int};$P_{hsp\text{-}90}$*::GFP; sid-1(pk3321);unc-119p::SID-1* or **(H)** *HSP-90::RFP*^{muscle};$P_{hsp\text{-}90}$*::GFP;sid-1(pk3321);unc-119p::SID-1* animals allowing for neuron-specific RNAi and crossed into the genetic background of a *unc-31* deletion mutant *(unc-31(e928))* treated with control (EV) or *unc-13* RNAi. Error bars represent SEM of three independent experiments; n.s. = not significant. **(I)** GFP and *hsp-90* transcript levels in *HSP-90::RFP*^{neuro};$P_{hsp\text{-}90}$*::GFP, sid-1(pk3321);vha-6p::SID-1* during intestine-specific *asp-12* or *clec-41* RNAi. **(J)** GFP and *hsp-90* transcript levels in *HSP-90::RFP*^{int};$P_{hsp\text{-}90}$*::GFP;sid-1(pk3321);unc-119p::SID-1* during neuron-specific *asp-12* or *clec-41* RNAi. **(K)** Paralysis assays of *HSP-90::RFP*^{neuro};$A\beta_{(3\text{-}42)}$ treated with EV or *pha-4* RNAi compared to $A\beta_{(3\text{-}42)}$ animals.
Paralysis data represent SEM of 3 biological replicates (100 animals per biological replicate).
Statistical significance was determined by Wilcoxon matched pairs-signed rank test. $^{*}P < 0.05$.
